# Supplementary figures and images for: A sporulation signature protease is required for assembly of the spore surface layers, germination and host colonization in Clostridioides difficile
Source: PLoS Pathog. 2023 Nov 13;19(11):e1011741. doi: 10.1371/journal.ppat.1011741 (PMC10681294; doi:10.1371/journal.ppat.1011741)

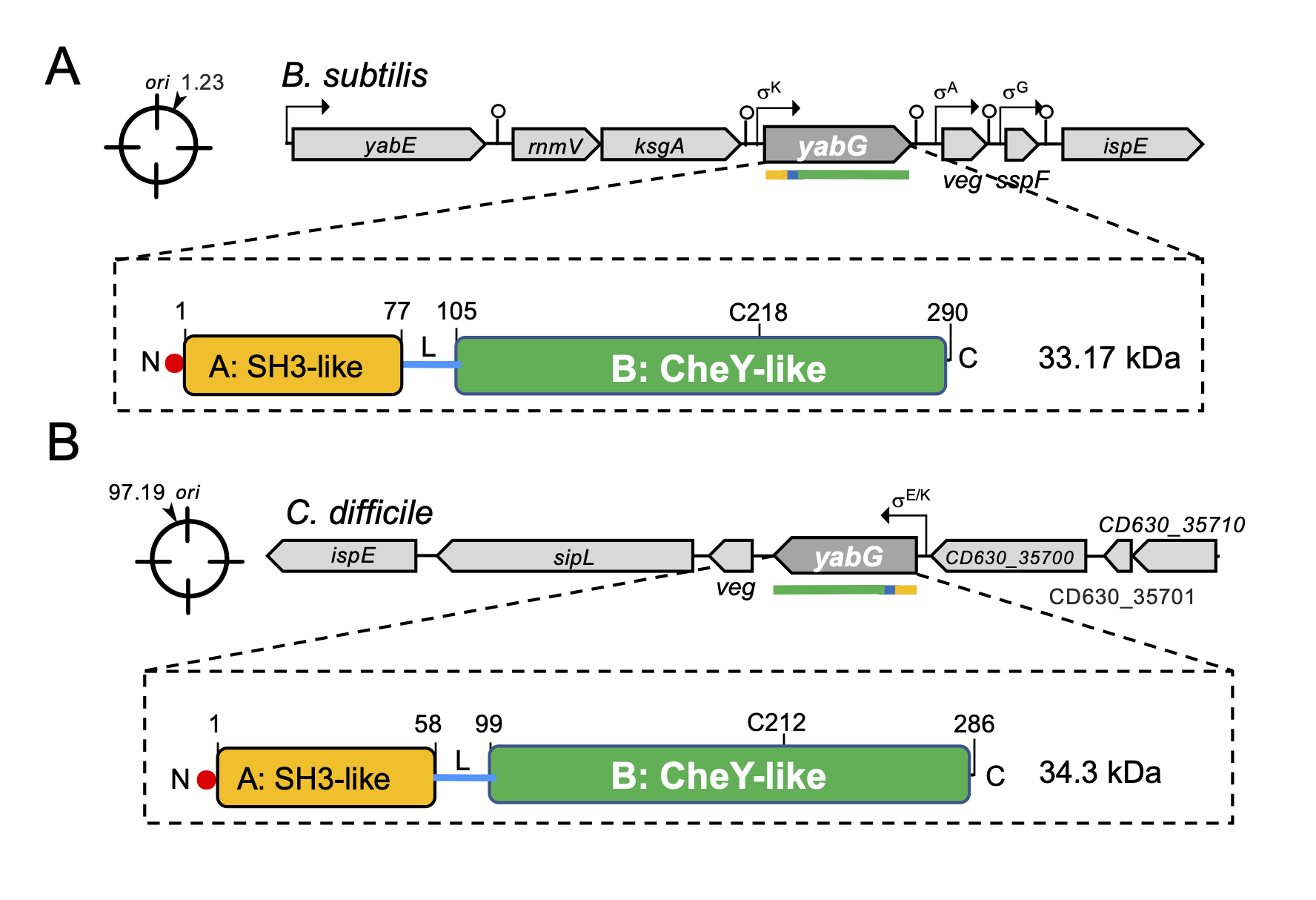

Supplement: S1 Fig — Schematic representation of the yabG region of the B. subtilis (A, at 1.23 map units; close and to the right to the origin of chromosome replication, ori) and C. difficile chromosomes (B, at 97.19 map units, close but to the left of ori). The bottom panel represents the predicted structural organization of the two proteins (see also Fig 1C). (TIFF) [file ppat.1011741.s001.tiff]

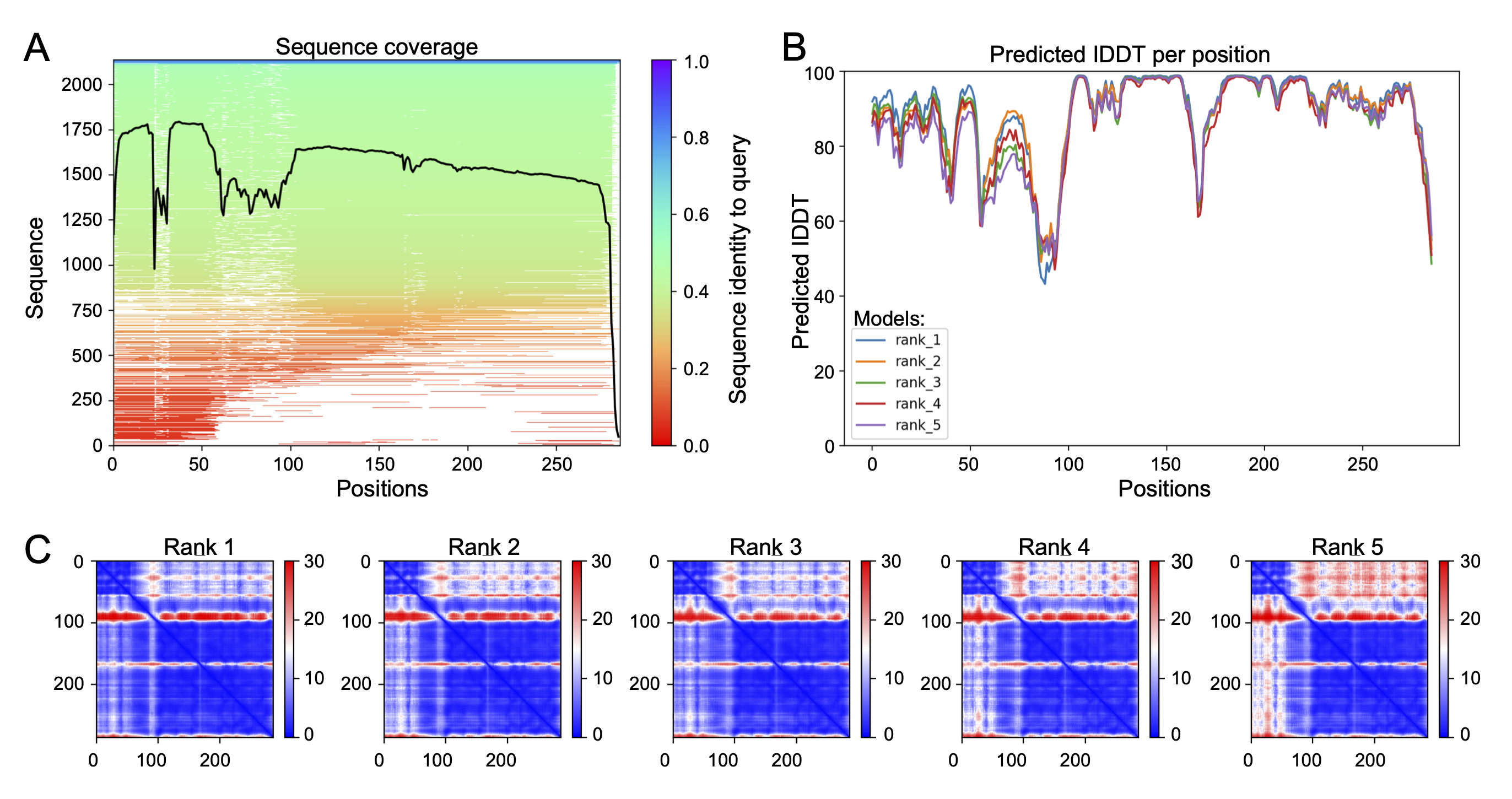

Supplement: S2 Fig — A: Sequence coverage; number of sequences and sequence identity to the query. B: Local Distance Difference Test (IDDT) per position. C: rank of the five models generated. The plots shown are reproduced from the output of the ColabFold server [105]. (TIFF) [file ppat.1011741.s002.tiff]

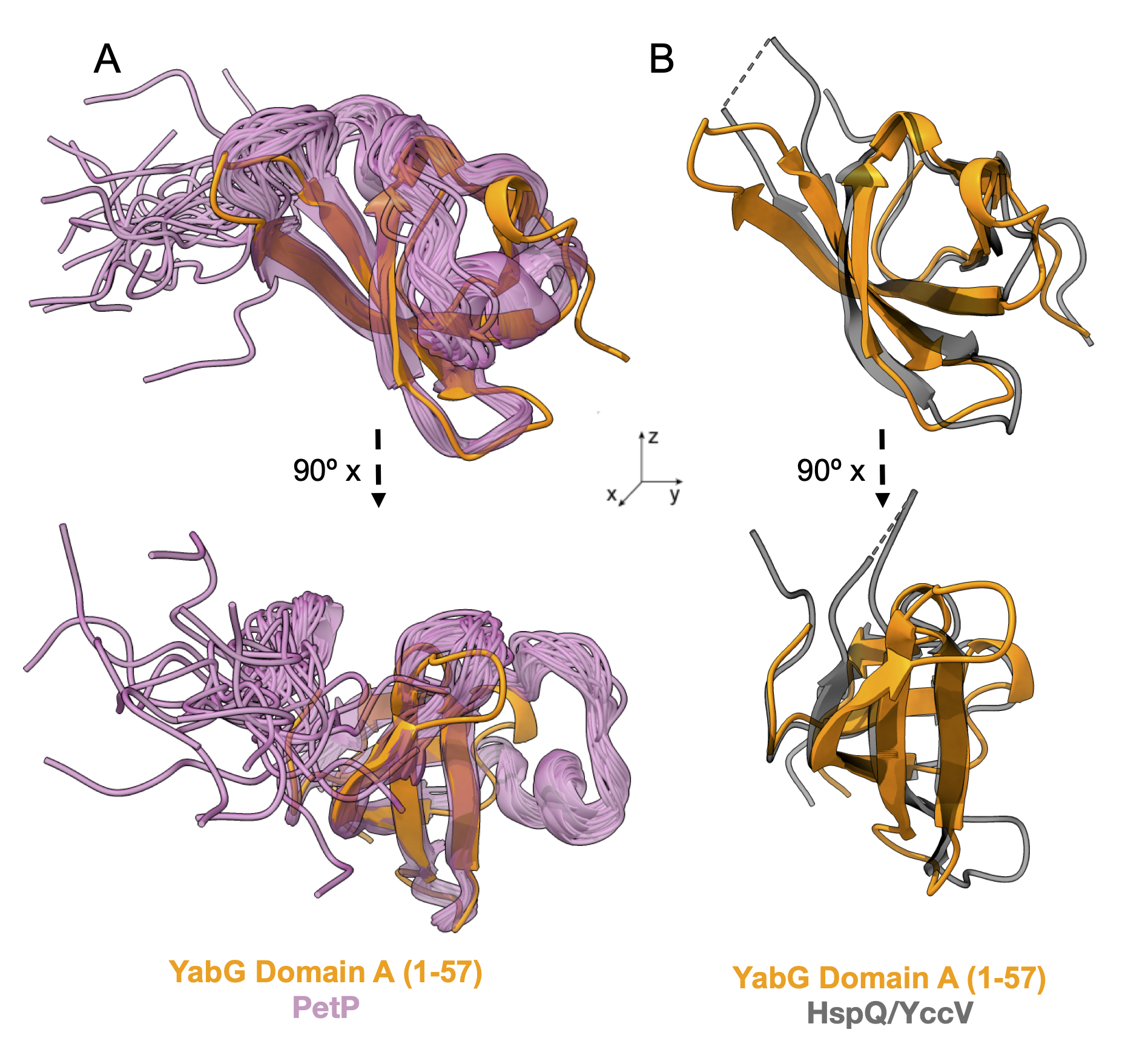

Supplement: S3 Fig — The AlphaFold2-generated model of the A domain of YabG (orange) is superimposed onto the NMR solution structure of the SH3 protein PetP a subunit of the cyanobacterial cytochrome b6f (A; pdb code 2n5u) and the crystal structure of E. coli HspQ (B; pdb code 5ycq; also known as YccV). Panel A shows the ensemble of the NMR structures determined for the PetP protein. (TIFF) [file ppat.1011741.s003.tiff]

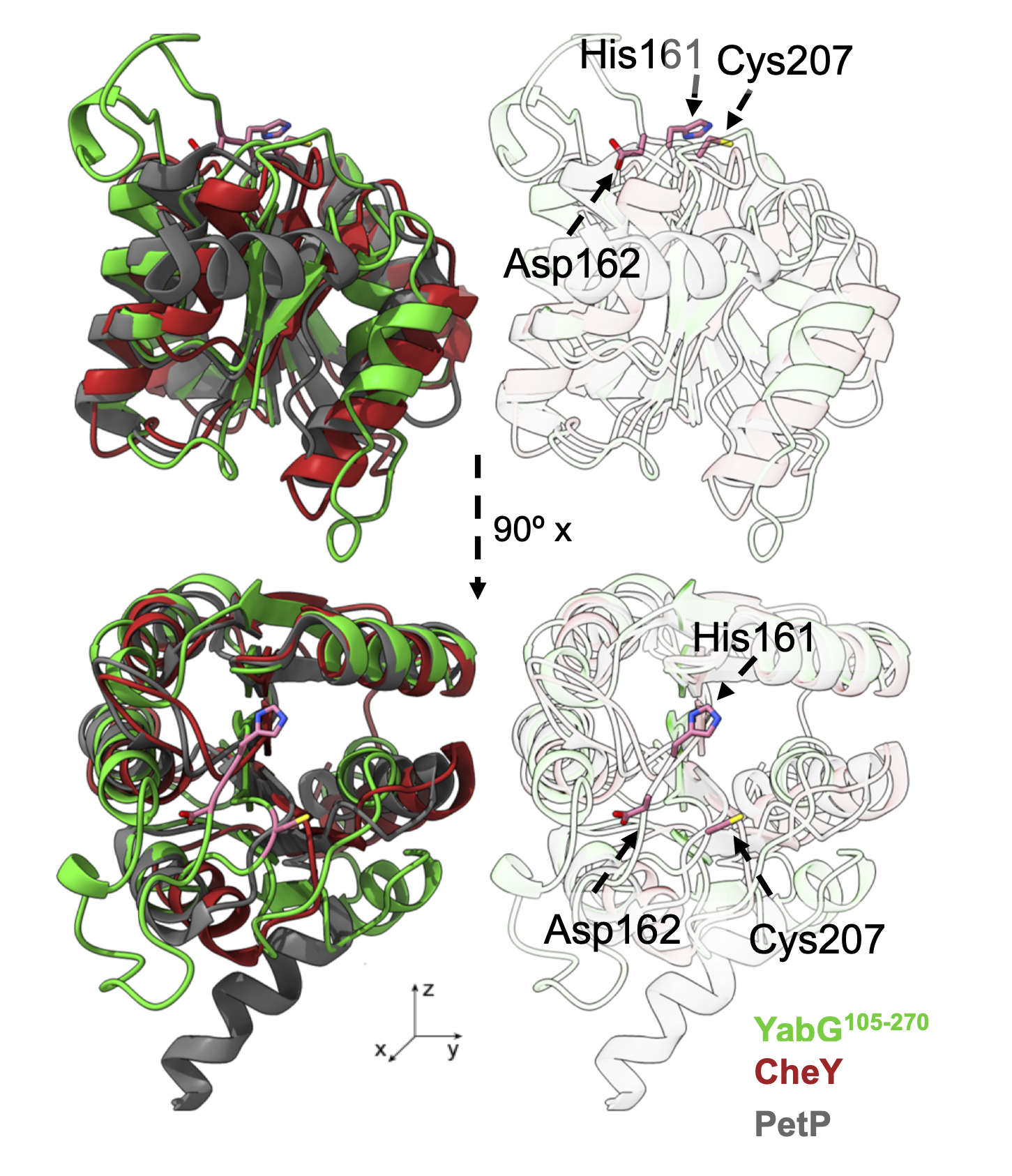

Supplement: S4 Fig — The AlphaFold2-generated model of YabG (green) is superimposed onto the crystal structures of CheY (pdb identifier: 5chy; brown) and KdpE of E. coli (pdb identifier: 4I85; black). The panels on the right show a trace of the structures to reveal the position of the His161, Asp162 and Cys207residues in YabG. (TIFF) [file ppat.1011741.s004.tiff]

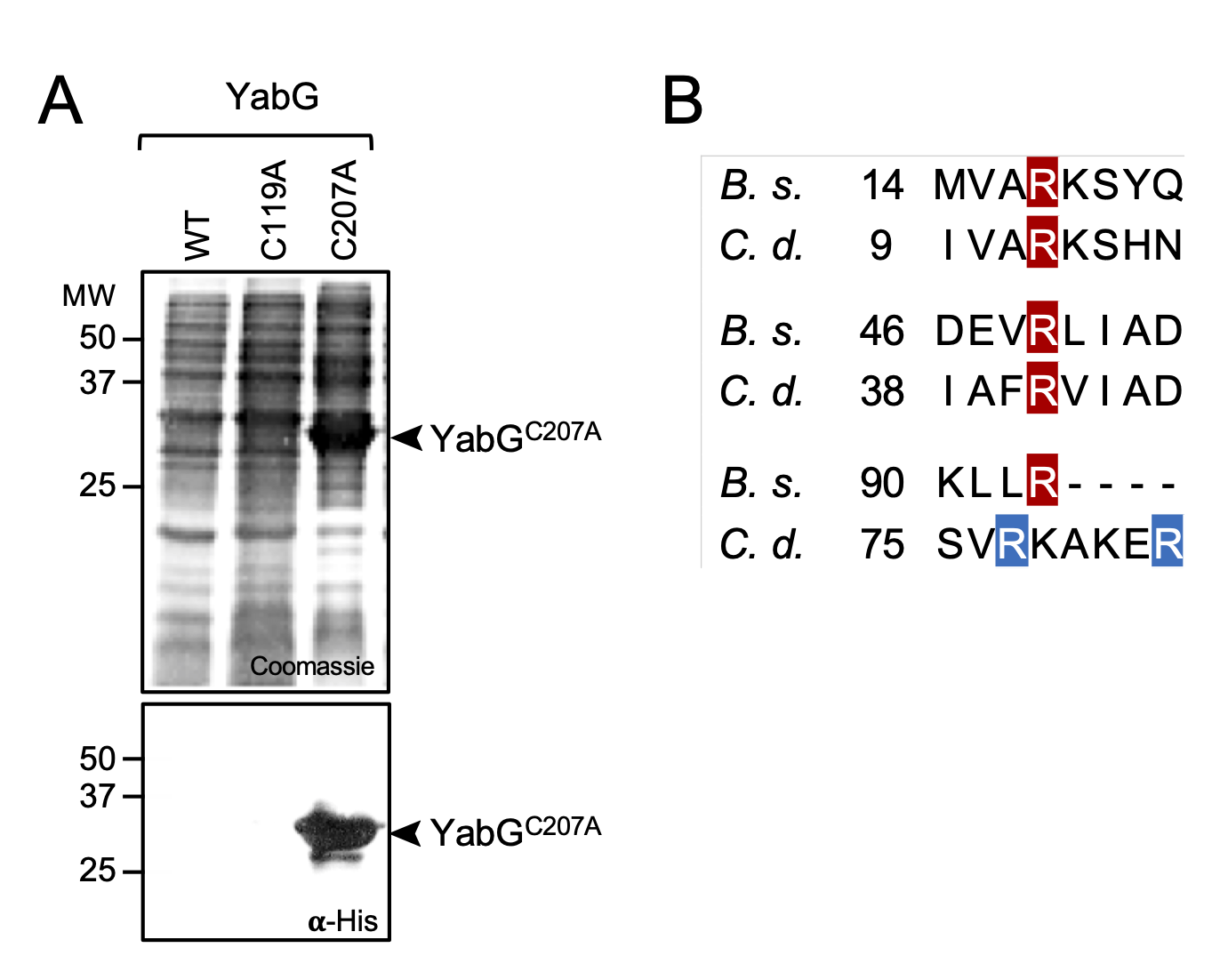

Supplement: S5 Fig — A: His10-YabGWT (WT), His10-YabGC119A (C119A) and His10-YabGC207A (C207A) were produced in E. coli. The proteins in whole cell extracts were resolved by SDS-PAGE and the gels stained with Coomassie or subject to immunoblotting with an anti-His6 antibody. B: The alignments show blocks of amino acids conserved between the YabG proteins of B. subtilis (B. s.) and C. difficile (C. d.) in the vicinity of the processing sites determined for the B. subtilis protein. Numbering is from the first residue of the proteins. (TIFF) [file ppat.1011741.s005.tiff]

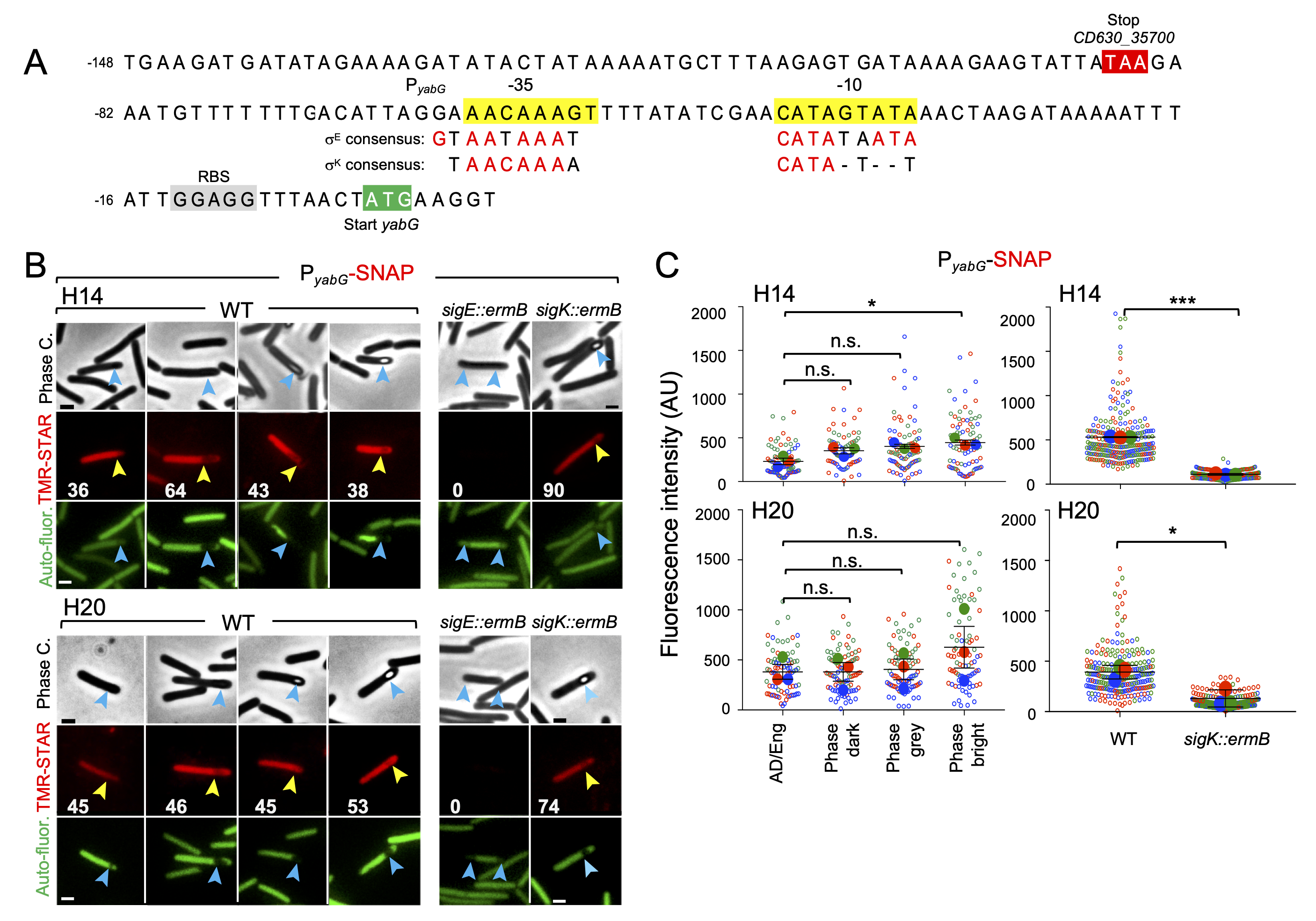

Supplement: S6 Fig — A: The panel shows the yabG regulatory region and highlights the putative -10 and -35 promoter elements with the consensus for σE/K binding indicated below (bases that match the consensus are in red; [76]). The ribosome binding site (RBS), the yabG start codon and the stop codon of the CD630_35700 gene are also indicated. B: Expression of a PyabG-SNAPCd transcriptional fusion in sporulating cells of the WT (630Δerm) and in congenic sigE and sigK mutants. The cells were collected after 14h or 20h of growth in 70:30 agar plates [53], stained with the TMR-Star SNAP substrate and examined by phase contrast and fluorescence microscopy. The blue arrowheads indicate the position of the forespore in the phase contrast and in the autofluorescence images (green channel); the yellow arrows show the mother cell-specific SNAPCd-TMR-Star signal (red). Note the disporic sporangium in the sigE mutant. The numbers indicate the percentage of sporangia at the indicated stages showing PyabG-SNAPCd expression. A representative stage for each mutant was selected. At least 50 cells were scored for each strain in each of three independent experiments. Scale bar, 1μm. C: Intensity of the fluorescence signal per cell for the PyabG-SNAPCd fusion in the mother cell during asymmetric division and engulfment (AD/Eng), and in sporangia of phase-dark, phase-grey or phase-bright forespores in the WT (top) or in sporangia of phase-grey and phase-bright forespores in a sigK::ermB mutant (bottom). No signal was detected in sigE::ermB sporangia. Fluorescence intensity is shown in arbitrary units (AU). SuperPlots were used to represent the data from three biological replicates; each dot corresponds to one cell, color-coded by experiment. The large circles represent the means from each experiment which were used to calculate the mean and standard error of the mean (horizontal lines) for the ensemble of the three experiments. Statistical analysis was carried using a Student’s t-test (right panels) or an o [file ppat.1011741.s006.tiff]

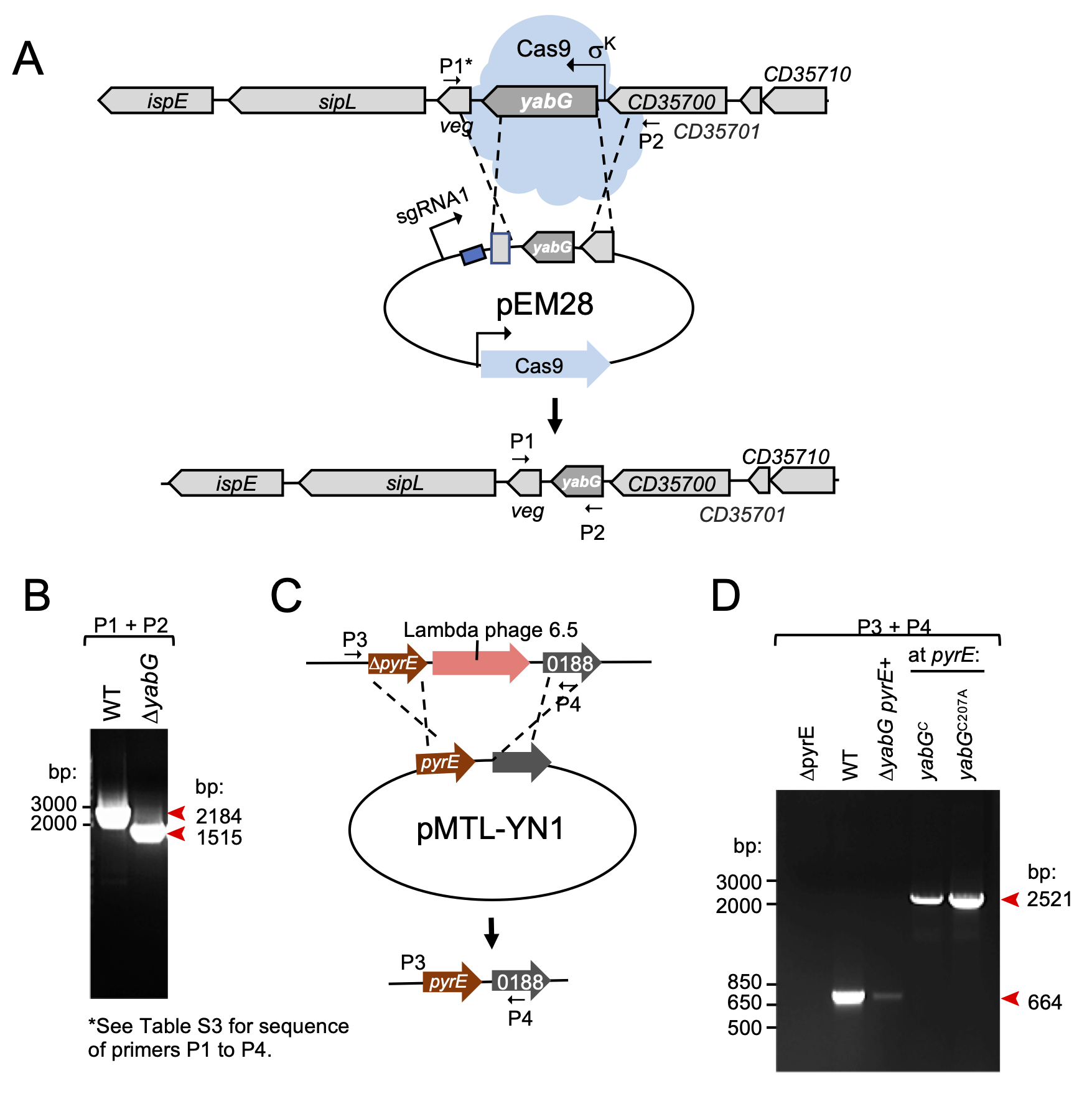

Supplement: S7 Fig — A: Genetic organization of the C. difficile chromosome in the vicinity of yabG. Plasmid pEM28 codes for the single guide RNA carrying the seed region of 20 nucleotides that directs Cas9 to yabG [95,108]. The position of the sequences recognized by primers P1 and P2 is also shown. B: Chromosomal DNA was prepared from the WT and a thiamphenicol resistant C. difficile conjugant and screened by PCR. The presence of the PCR product of 1515 bp, as opposed to 2184 bp for the WT, identifies the yabG in-frame deletion mutation. C: Schematic representation of ΔpyrE reversion using homologous recombination between pMTL-YN1 and the genome of 630ΔermΔyabGΔpyrE. D: ΔyabG pyrE+ isolates was screened by PCR for the presence/absence of a reverted pyrE gene. The ΔyabG pyrE+ strain results from recombination of pMTL-YN1 at the pyrE locus (C). In trans complementation of the ΔyabG in-frame deletion was accomplished by introducing the WT copy of yabG (yabGC) at the pyrE locus using allelic exchange according to the scheme in C using pEM39; similarly, but using pEM41, the yabGC207A allele was transferred to the pyrE locus (see also the Material and Methods section). (TIFF) [file ppat.1011741.s007.tiff]

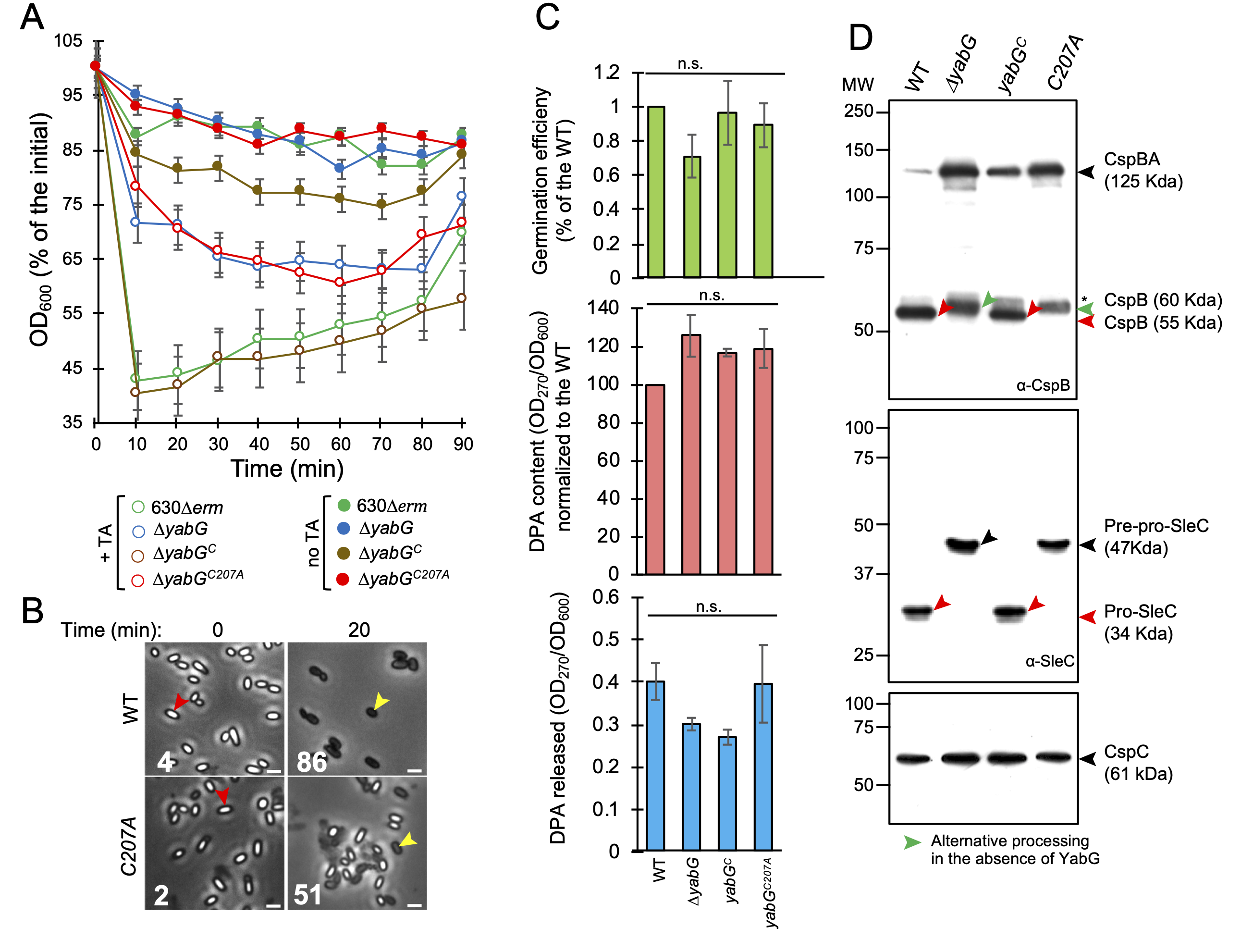

Supplement: S8 Fig — A: Purified spores were heat activated and BHI-TA (0.5%) was added (open symbols). Germination was followed by the decrease in the optical density of the spore suspension at 600 nm and expressed as the percentage of the initial OD600. In control experiments, the spores were maintained in BHI, with no TA (closed symbols). B: Phase contrast of purified spores incubated in BHI in the presence of 0.5% TA. The numbers refer to the percentage of phase-dark spores. Scale bar, 1 μm. C: Top panel: germination efficiency for spores of the indicated strains, expressed as the fraction of the WT; middle panel: the DPA released after TA-induced germination at 37°C is shown for spores of the indicated strains and it is expressed as the ratio between the OD270 and the initial OD600 of the spore suspension; bottom panel: spores resuspended in PBS supplemented with 0.5% TA were boiled to determine the total DPA content (total DPA released; blue bars), expressed as a percentage of DPA content of the WT; The results are the average of three independent experiments; statistical significance was determined using ANOVA and Tukey’s test. D: Immunoblotting of proteins extracted from density-gradient purified spores produced by the WT, ΔyabG, yabGC207A and the complementation strain (yabGC). The proteins were resolved by SDS-PAGE and the gels subject to immunoblot analysis with anti-CspB, anti-SleC and anti-CspC antibodies. The experiments were repeated at least three times. (TIFF) [file ppat.1011741.s008.tiff]

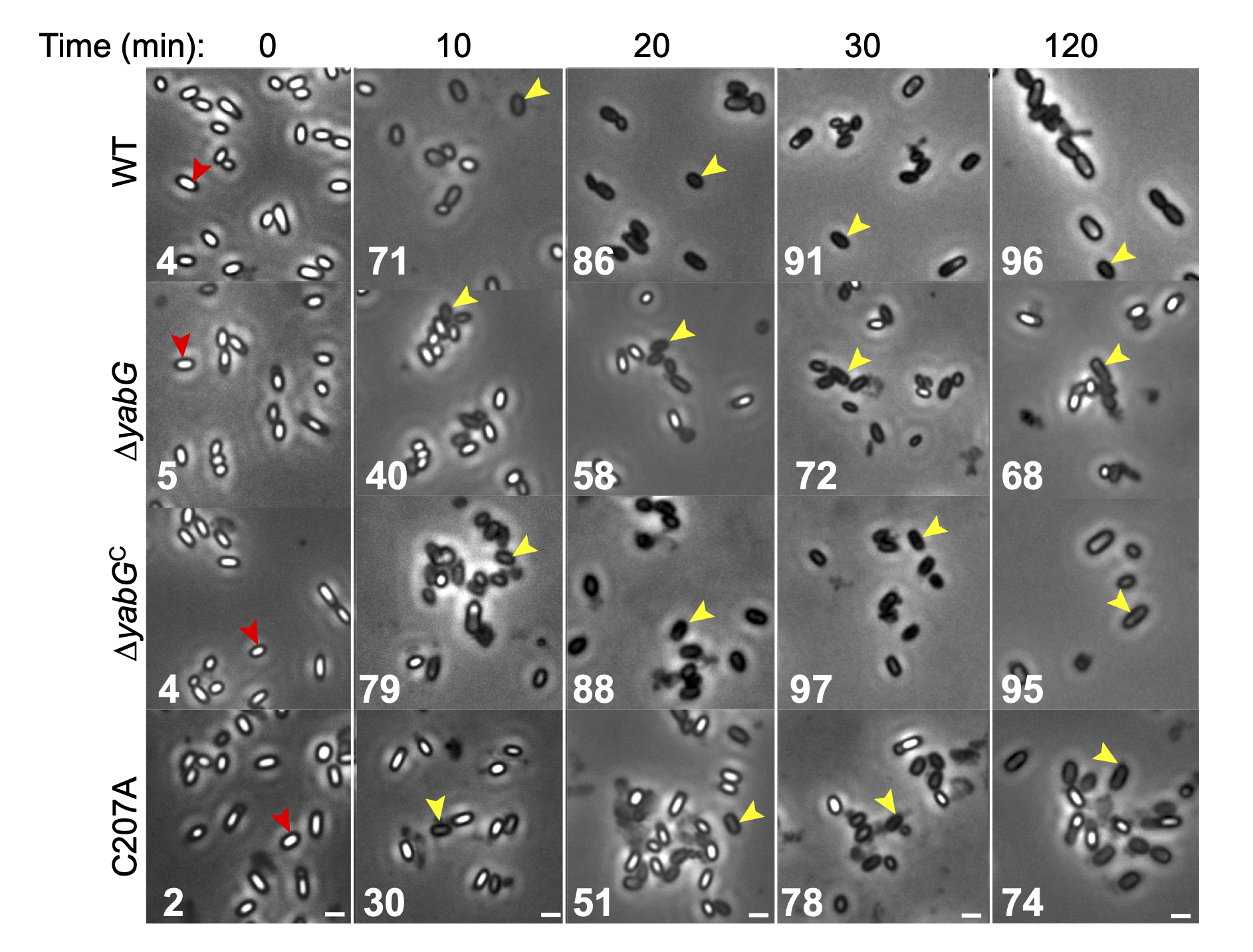

Supplement: S9 Fig — ΔyabG, yabGC207A, yabGC, and WT highly purified spores were heat activated for 10 min at 80°C and TA added (to 0.5%) in rich media under anaerobic conditions. Samples were collected at the indicated times (in min) after TA addition and imaged by phase contrast microscopy. Red arrowheads indicate phase-bright free spores while yellow arrowheads show phase-dark, germinating spores. Numbers refer to the percentage of phase-dark spores scored at the indicated times: The data refers to one of three independent experiments. Scale bar 1 μm. (TIFF) [file ppat.1011741.s009.tiff]

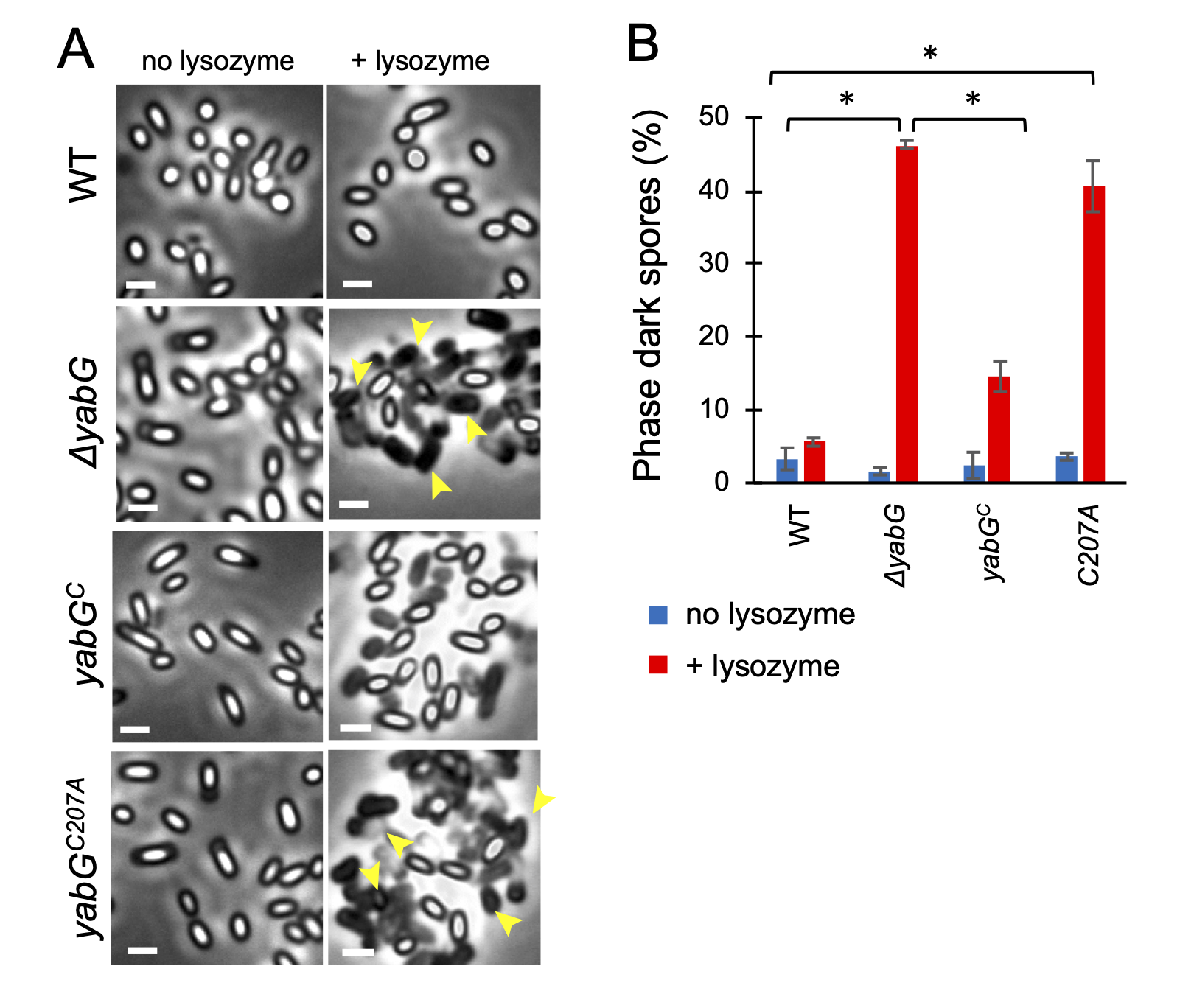

Supplement: S10 Fig — A: Purified spores of the indicated strains were incubated for 30 min at 37°C in the absence or in the presence of lysozyme (250 μg/ml). Following incubation, the samples were examined by phase contrast microscopy (left panel) and the percentage of phase-dark spores scored (right panel). The yellow arrowheads point to phase dark-spores. Scale bar, 1 μm. B: Shows the percentage of phase-dark spores before and after treatment with lysozyme 250 μg/ml. The results are the average for three independent experiments; 170–250 spores were counted per each strain in each experiment. The p-value is indicated for all comparisons whose differences were found to be statistically significant using ANOVA and Tukey’s test (*, p≤ 0.05). (TIFF) [file ppat.1011741.s010.tiff]

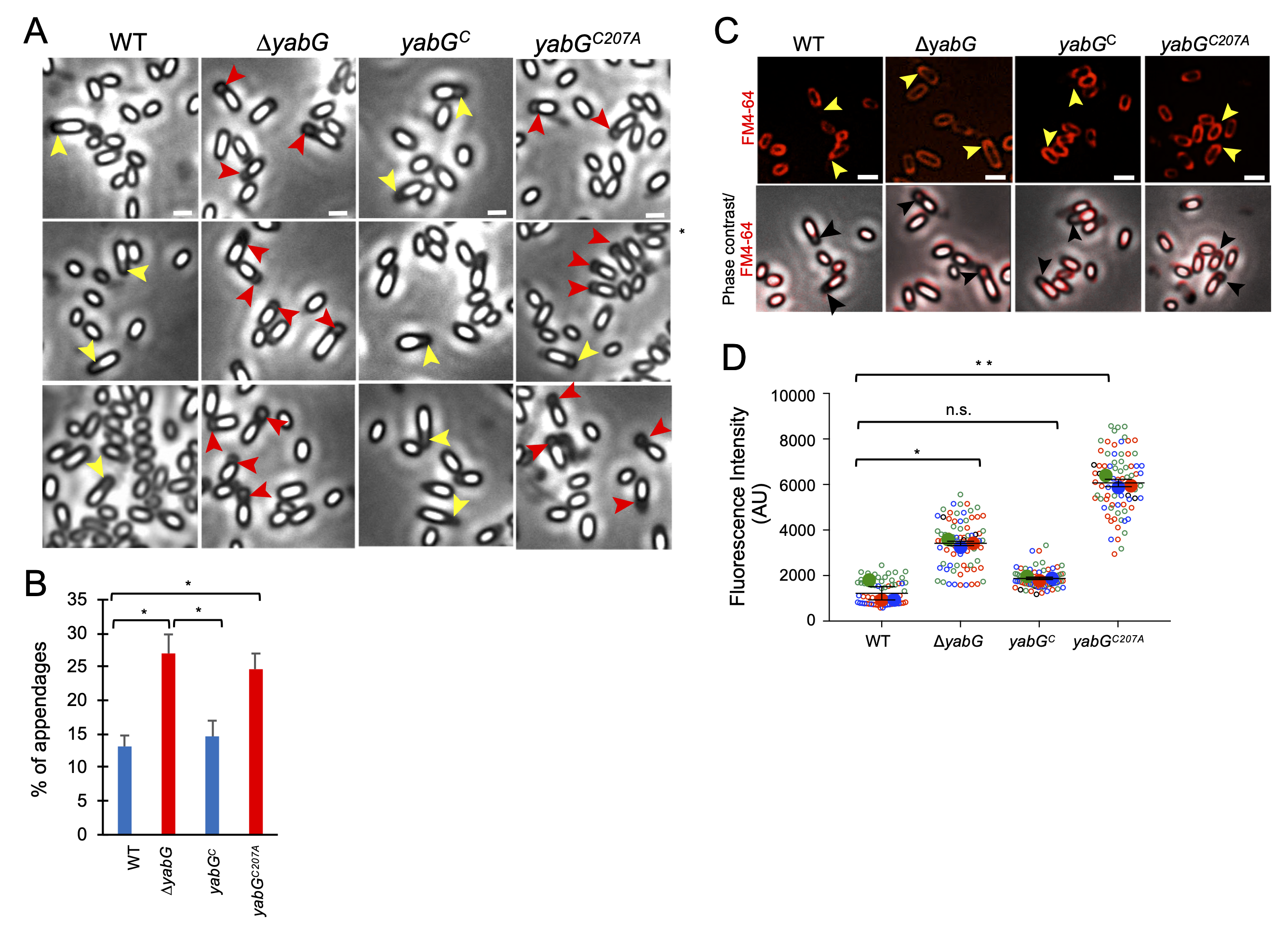

Supplement: S11 Fig — A: Density gradient purified spores of the indicated strains (WT, yabGC, ΔyabG and yabGC207A) were examined by phase contrast and fluorescence microscopy. The figure shows three panels, aligned vertically, for spores of each strain. Yellow arrowheads, polar appendages in the WT of yabGC strain; red arrowheads, the squarish appendage in the two yabG mutants. Scale bar, 1 μm. B: Percentage of spores with appendages for each of the indicated strains. Spores were considered to possess an appendage if its length was ≥ 0.25 μm. At least 60–80 spores were scored for each strain; three biological replicates were performed. Statistical significance was determined using ANOVA and Tukey’s test (* p< 0.05). C: Spores of the indicated strains were stained with FM4-64 and imaged by phase contrast and fluorescence microscopy. The arrowheads (yellow in the fluorescence images and black in the phase contrast images, point to the appendage region). Scale bar, 1 μm. D: Quantification of the FM4-64 intensity signal associated with the appendage region of purified spores of WT, ΔyabG, yabGC and yabGC207A strains. Note that the staining of the appendage with FM4-64 is higher for spores of the ΔyabG and yabGC207A mutants compared to the WT and yabGC spores. For each strains, 30–50 spores were scored from three independent experiments. SuperPlots were used to represent the data; each dot corresponds to one cell, color-coded by experiment. The large circles represent the means from each experiment which were used to calculate the mean and standard error of the mean (horizontal lines) for the ensemble of the three experiments. Statistical analysis was carried out using using ANOVA and Tukey’s test. *, p<0.05; **, p< 0.001. (TIFF) [file ppat.1011741.s011.tiff]

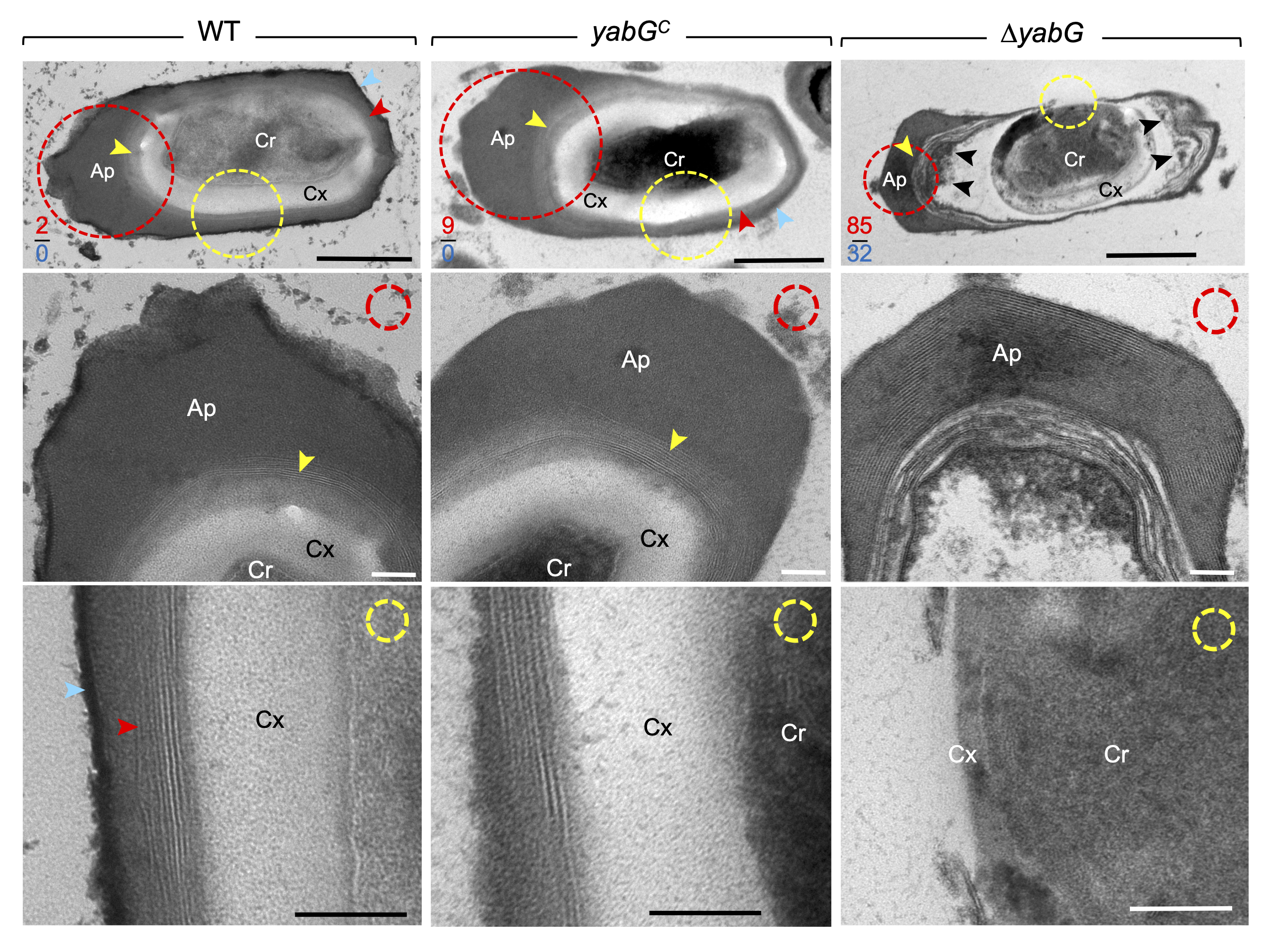

Supplement: S12 Fig — Purified spores of the WT (630Δerm), ΔyabG mutant and the complementation strain (yabGC) were analysed by thin sectioning TEM. Red arrowheads point to the coat region, blue arrowheads to the electron dense exosporium and yellow arrowheads to the region of transition between the coat and the appendage. The regions delimited by the red and yellow circles in the top panels are magnified on the bottom panels, as indicated. Cr, spore core; Cx, cortex; Ap, appendage region. The numbers refer to the percentage of spores in which the coat is detached from the cortex (red) or which show a prominent polar appendage, with a lamellar structure (blue) (see also Fig 2C). Between 60–95 spores were scored for each strain in two independent experiments. Scale bar, 500 nm (top panels) and 100 nm (all other panels). (TIFF) [file ppat.1011741.s012.tiff]

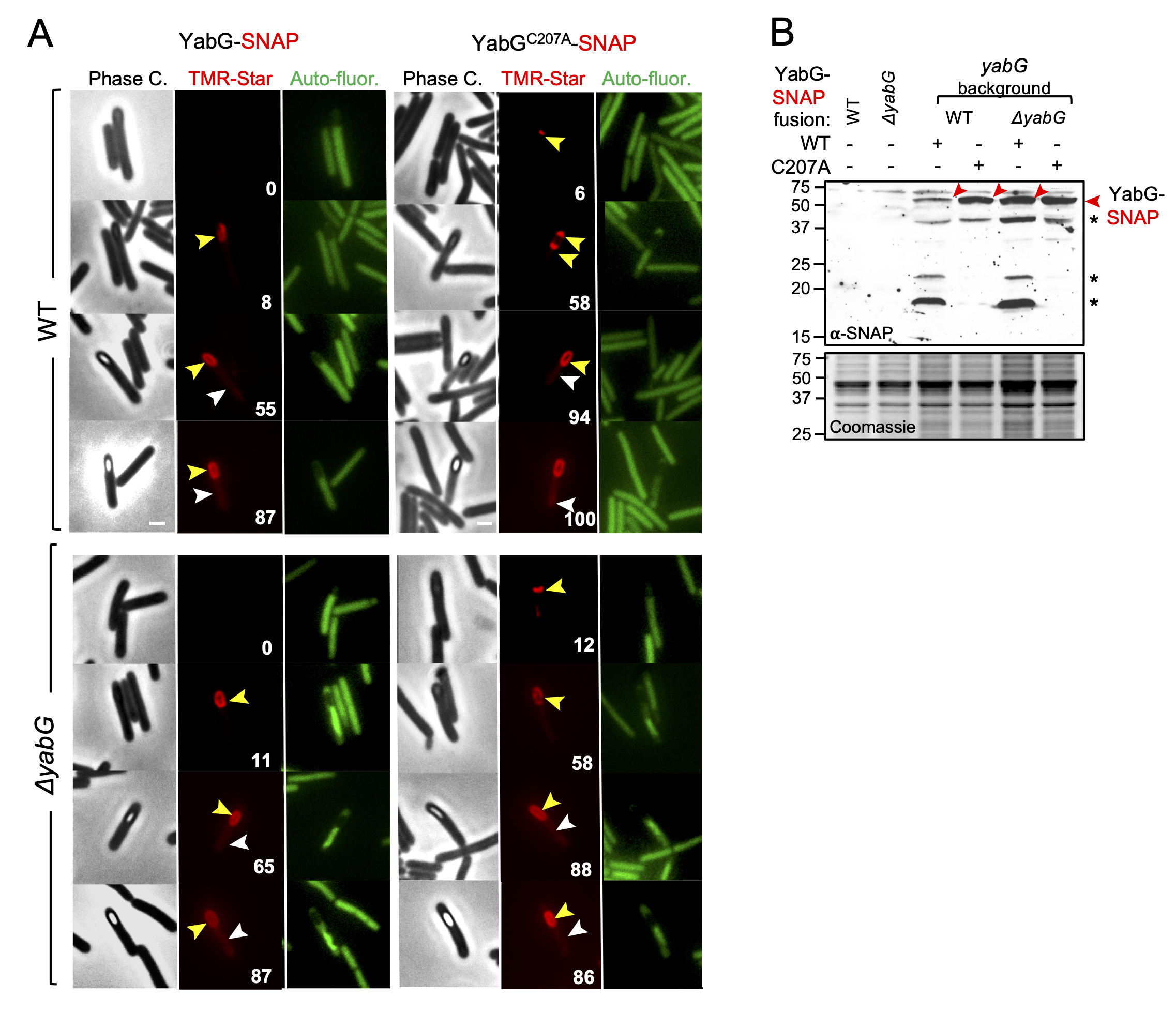

Supplement: S13 Fig — A. Localization of YabGWT-SNAPCd and YabGC207A-SNAPCd in the WT and in the ΔyabG mutant. Cells were collected after 14h of growth in 70:30 agar plates, stained with the SNAP substrate TMR-Star and examined by phase contrast and fluorescence microscopy (red channel for the TMR signal and the green channel for autofluorescence signal). The numbers refer to the percentage of cells at the represented stage exhibiting SNAP fluorescence. The data shown are from one experiment of three independent experiments. For each strain, at least 145 cells were scored per time point. Scale bar, 1 μm. B: Accumulation of YabG-SNAPCd and YabGC207A-SNAPCd in sporulating cells at 14h in the WT strain 630Δerm, and in the ΔyabG mutant. Whole cell extracts were prepared, the proteins resolved by SDS-PAGE and the gels subject to immunoblotting with anti-SNAP antibodies. The red arrowhead point to the position of YabGC207A-SNAP (52 kDa) and asterisks indicate possible degradation products that include the SNAP moiety (~19.4 kDa). Asterisks show the position of degradation products or cross-reactive species. (TIFF) [file ppat.1011741.s013.tiff]

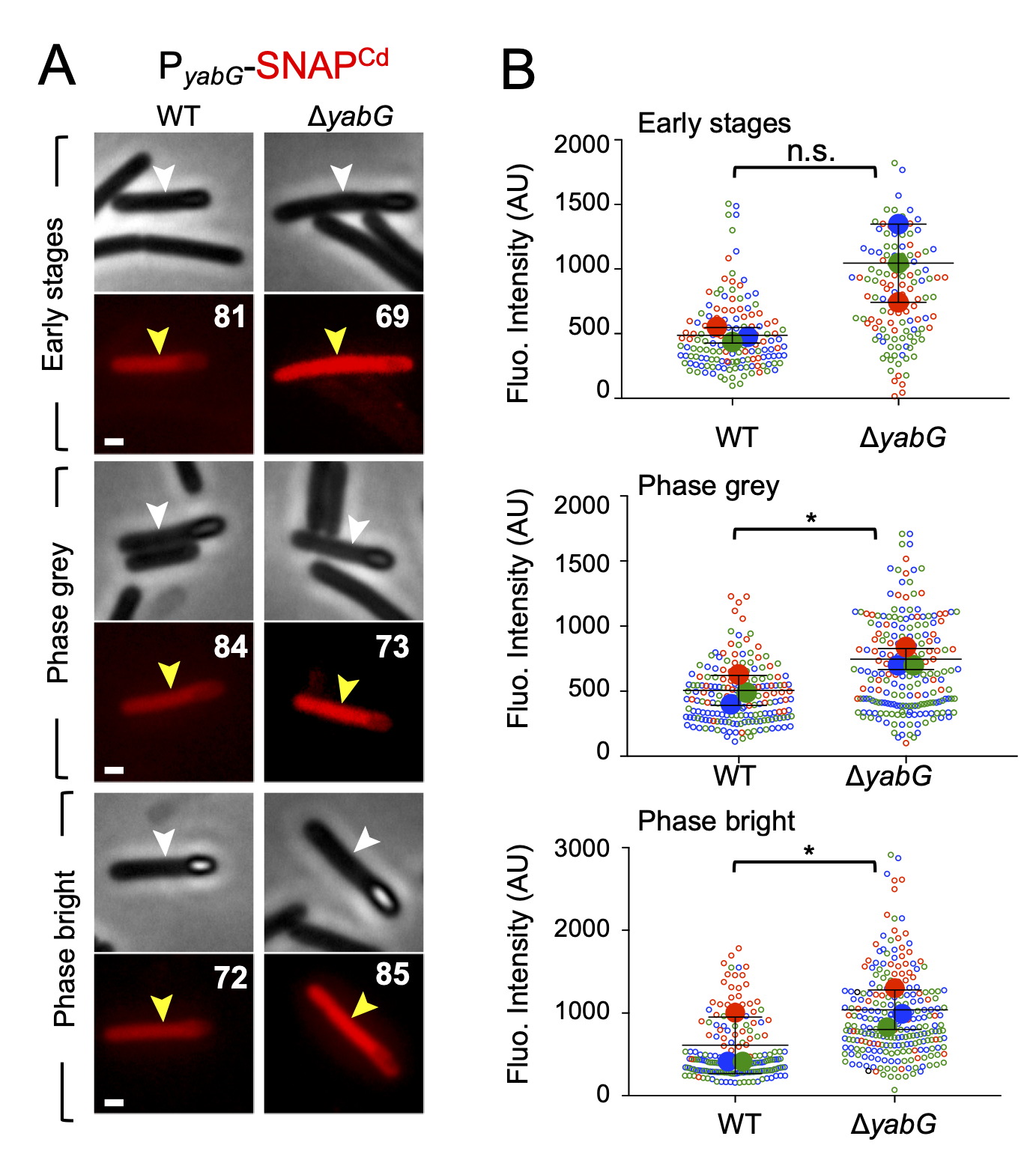

Supplement: S14 Fig — A: Samples from cultures of the WT, and congenic ΔyabG and yabGC207A mutants expressing a PyabG-SNAPCd transcriptional fusion were collected after 20h of growth in 70:30 agar plates and labelled with TMR-Star and imaged by phase contrast and fluorescence microscopy. The numbers in the panels show the percentage of sporangia with signal from the SNAPCd-TMR complex. Expression of the PyabG-SNAPCd fusion was scored in early stage sporangia (most with phase-dark spores), and in sporangia of phase-grey and phase-bright spores. White arrowheads, mother cell in the phase contrast images; yellow arrowheads, signal from SNAPCd-TMR. Scale bars, 1 μm. B: Shows the distribution of the fluorescence signal (in arbitrary units, AU) in the different types of sporangia considered. For each stage, 50 sporangia were scored, per strain in each of three biological replicates. SuperPlots were used to represent the data, with each dot corresponding to one cell and the three experiments shown with different colors. The large circles represent the means from each experiment and were used to calculate the mean and standard error of the mean (horizontal lines) for the collective of the three experiments. Statistical analysis was carried out using a Student’s t-test. * indicates p<0.05. Scale bar, 1 μm. (TIFF) [file ppat.1011741.s014.tiff]

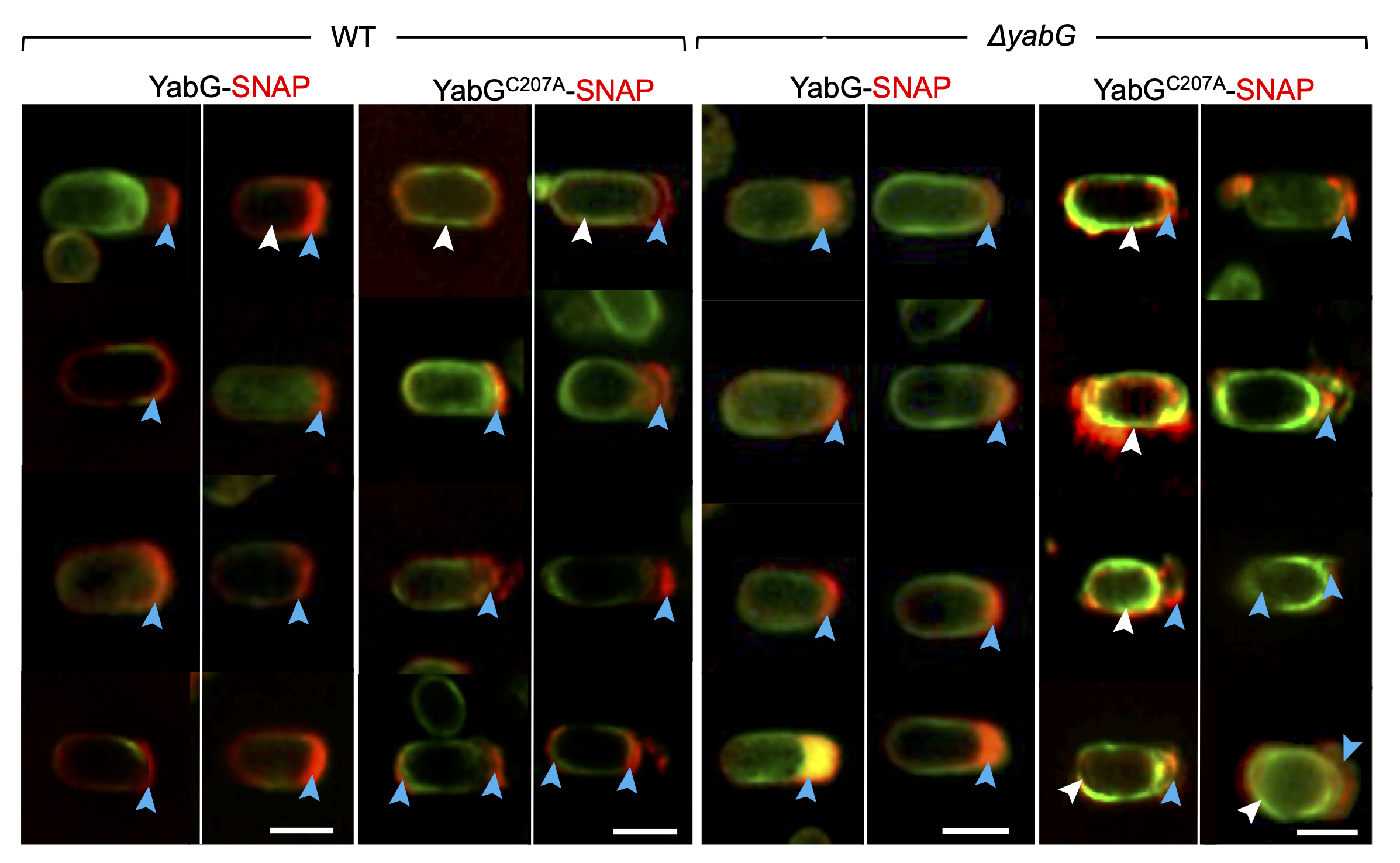

Supplement: S15 Fig — Localization of YabGWT- or YabGC207A-SNAPCd in mature spores using super resolution structured illumination microscopy (SR-SIM), in either the WT or ΔyabG backgrounds. The spores were stained with the membrane dye MTG (green) and with TMR-Star (red) prior to imaging. The blue arrowheads point to the appendage-associated signal; the white arrowheads point to the signal in other regions of the spore (see also Fig 6A). Scale bar, 500 nm. (TIFF) [file ppat.1011741.s015.tiff]
