# Supplementary material for: A sporulation signature protease is required for assembly of the spore surface layers, germination and host colonization in Clostridioides difficile
Source: PLoS Pathog. 2023 Nov 13;19(11):e1011741. doi: 10.1371/journal.ppat.1011741 (PMC10681294; doi:10.1371/journal.ppat.1011741)
Supplement: S1 Table — (PDF) [file ppat.1011741.s016.pdf]

Supplemental Tables

S1 Table - Bacterial strains.

Strain	Relevant genotype/properties	Origin/Reference
E. coli		
DH5α	General molecular cloning host	Invitrogen
BL21 (DE3)	Host for protein production	Novagen
HB101 (RP4)	Host for conjugation in *C. difficile*	Laboratory stock
AHCD 494	BL21 (DE3) (pEM6)	This study
AHEC 537	BL21 (DE3) (pEM12)	“
AHEC 561	BL21 (DE3) (pEM13)	“
AHEC 643	BL21 (DE3) (pEM23)	“
AHEC 646	BL21 (DE3) (pEM23+pEM6)	“
AHEC 647	BL21 (DE3) (pEM23+pEM12)	“
AHEC 652	BL21 (DE3) (pEM21)	“
AHEC 645	BL21 (DE3) (pEM24)	“
AHEC 703	BL21 (DE3) (pEM38)	“
AHEC1021	HB101 (pRP4) (pSR77)	“
C. difficile		
630Δ*erm*	wild type	[94]
AHCD 772	630Δ*erm* Δ*pyrE*	“
AHCD 535	630Δ*erm sigK::erm*	[29]
AHCD 533	630Δ*erm sigE::erm*	“
AHCD 817	630Δ*erm* (pCAF3)	“
AHCD 1203	630Δ*erm* Δ*yabG*	“
AHCD 1204	630Δ*erm* Δ*yabG*^{C}	“
AHCD 1205	630Δ*erm* Δ*yabG*^{C207A}	“
AHCD 1150	630Δ*erm* Δ*yabG* Δ*pyrE*	“
AHCD 974	630Δ*erm* (pEM5)	“
AHCD 1193	630Δ*erm* (pEM40)	“
AHCD 1214	630Δ*erm* Δ*yabG* (pEM5)	“
AHCD 1215	630Δ*erm* Δ*yabG* (pEM40)	“
AHCD 1382	630Δ*erm* (pEM7)	“
AHCD 1357	630Δ*erm* Δ*yabG* (pEM7)	“
AHCD 1461	630Δ*erm sigK::erm* (pEM7)	“
AHCD 1470	630Δ*erm sigE::erm* (pEM7)	“
AHCD 1380	630Δ*erm* Δ*yabG*^{C207A} (pEM7)	“
AHCD 1502	630Δ*erm* (pSR77)	“
AHCD 1503	630Δ*erm*Δ*yabG* (pSR77)	“
AHCD 1504	630Δ*erm* Δ*yabG pyrE::*Δ*yabG*^{C207A} (pSR77)	“
AHCD 1465	630Δ*erm* (pCAF3)	“
AHCD 1466	630Δ*erm* Δ*yabG pyrE::*Δ*yabG*^{C207A} (pCAF3)	
