# Supplementary material for: A sporulation signature protease is required for assembly of the spore surface layers, germination and host colonization in Clostridioides difficile
Source: PLoS Pathog. 2023 Nov 13;19(11):e1011741. doi: 10.1371/journal.ppat.1011741 (PMC10681294; doi:10.1371/journal.ppat.1011741)
Supplement: S2 Table — (PDF) [file ppat.1011741.s017.pdf]

S2 Table – Plasmids used in this study.

Plasmid	Relevant features	Origin
pET16b	His-tag fusion protein production vector (Amp^R)[a]	Novagen
pACYCDuet-1	Vector for the co-expression of two genes (Cm^R)	"
pMTL84121	*Clostridium* modular plasmid (Cm^R/Tm^R)	[100]
pMTL-YN1	Plasmid for *pyrE* reversion through ACE (Cm^R/Tm^R)	[94]
pMTL-YN1C	Plasmid for complementation through ACE (Cm^R/Tm^R)	"
pMTL431521	CRISPR-Cas9 genome editing vector (Cm^R/Tm^R)	[95]
pFT58	pMTL84121-linker-$SNAP^{Cd}$ (Cm^R/Tm^R)	[29]
pSR77	P_{cotE}-*cotA* in pMTL84121 (Ap^R/Tm^R)	This work
pCAF3	P_{cotE}-*cdeM* in pMTL84121 (Ap^R/Tm^R)	"
pEM5	*yabG* in pFT58	"
pEM6	his_{10}-*yabG* in pET16b	"
pEM7	P_{yabG}-$SNAP^{Cd}$ in pMTL84121	"
pEM12	his_{10}-$yabG^{C207A}$ in pET16b	"
pEM13	his_{10}-$yabG^{C119A}$ in pET16b	"
pEM21	his_{10}-$yabG^{D162A}$ in pET16b	"
pEM23	*cspBA-strep*-tag in pACYC-duet1	"
pEM24	his_{10}-$yabG^{D248A}$ in pET16b	"
pEM28	Δ*yabG* in pMTL431521	"
pEM38	his_{10}-$yabG^{H161A}$ in pET16b	"
pEM39	Δ$yabG^{C}$ in pMTLYNC	"
pEM40	$yabG^{C207A}$ in pFT58	"
pEM41	Δ$yabG^{C207A}$ in pMTLYN1C	"

[a]Resistance to ampicillin (Amp^R), cloranfenicol (Cm^R) and tianfenicol (Tm^R).
