# Supplementary material for: A sporulation signature protease is required for assembly of the spore surface layers, germination and host colonization in Clostridioides difficile
Source: PLoS Pathog. 2023 Nov 13;19(11):e1011741. doi: 10.1371/journal.ppat.1011741 (PMC10681294; doi:10.1371/journal.ppat.1011741)
Supplement: S3 Table — (PDF) [file ppat.1011741.s018.pdf]

S3 Table – Oligonucleotides used in this study.

Primer	Sequence (5' to 3')[a]
P1	AGTGGTACATAAGATGAATGAATTGGG
P2	CGTTTATATCTACTTTTCCGTCTAC
P3	CAATAATTTTATAACATTAACATGG
P4	GTGTTACTTAAAAAATGTAAAT
P5	TTTTCGTCGACATTGGTGGTTCTGAAACAAGGTTTTAGAGCTAGAAATAGCAAGTTAAA<br>ATAAGGCTAGTCCGTTATCAACTTGAAAAAGTGGCACCGAGTCGGTGC
P6	CGCGCGCGGCGATCGCATAAAAATAAGAAGCCTGCAAATGCAGGCTTCTTATTTTTATA<br>AAAAA<br>AGCACCGACTCGGTGCCACTTTTTCAAGTTG
P7	CCCCGGCGCGCCTGGTGGATTTTATGAAATTG
P8	GTCATCAATATAAGCATCTGCTATTAC
P9	GTAATAGCAGATGCTTATATTGATGACGGAAAATTTAGATGGGCAATGCCAAAAAC
P10	ACTGCGATCGCGCAAAGTATCTTCCCATCTGC
P11	CCGCTCGAGAGTGGTACATAAGATGAATGAATTGGG
P12	CGGGATCCGAACAGTAGCCACAATATCAC
P13	CATAATTTGATTGGGCTGCTCCTGCG
P14	CGCAGGAGCAGCCCAATCAAATTATG
P15	CCGGAATTCGGGTCAGATTTTGATGG
P16	ATTTCACAATCTTTATCCATAGTTAAACCTCC
P17	ATGGATAAAGATTGTGAAATGAAGAGAACC
P18	CCGCTCGAGTTACCCAAGTCCTGGTTTCCCCAAACG
P19	GCTGTCGACGCTAGAATAGACAATGAG
P20	CATTATAACATATCTCTCCC
P21	CCGGAATTCGAAATACGGGAGACCGTGTCTGG
P22	CATTTTTCATTGCCCTCCCTGTAAGCG
P23	GGGAGAGATATGTTATAATGGATAAAGATTGTGAAATGAAGAGAACC
P24	CCCAAGCTTACAGGGAGGGCAATGAAAAATGC
P25	GAGGGCAATGAAAAATGGATAAAGATTGTGAAATGAAGAGAACC
P26	GTAATGTAGAATTCCCTGCACCATTTG
P27	CACTCCCTCGAGATTTTAATTTAAATGTATAG
P30	AGCTGCGGATCCACCACCACCAAGATGTAATATTGTTTTTGGC
P31	CATGCCATGGTTATAATAAATTATGAATTAATTG
P32	TTGCGGCCGCTTATTTTTTCGAACTGCGGGTGGCTCCAAGCGCTTCTTAA<br>AACATCAAACATTCCTCTAAC
P33	CCGCTCGAGATGAAGGTAGGAGACATTG
P34	CGGGATCCCTAATGTAATATTGTTTTTGGC
P35	CCGCTCGAGATGCAATTTCAAATAGGGGATATGG
P36	CGGGATCCTTAATTGGACTTATAAGGCATACC
P37	GATAAAGAATATTTAAAAATTGCCTTGGATGTATATGC
P38	GCATATACATCCAAGGCAATTTTTAAATATTCTTTATC
P39	GTAATAACAGGTCATGCTGCAATGACAATAAAAAGAGG
P40	CCTCTTTTTATTGTCATTGCAGCATGACCTGTTATTAC
P41	GTAATAACAGGTGCTGATGCAATGACAATAAAAAGAGG
P42	CCTCTTTTTATTGTCATTGCATCAGCACCTGTTATTAC
P43	GCTTGTTCAAGAATTGCTGTAGTAGTTCCTATTG
P44	CAATAGGAACTACTACAGCAATTCTTGAACAAGC
P45	CCCAAGCTTCCCTGTCTATTGAGGAATTCGC
P46	CGGGATCCCGGTAAATTATGTATCATTATG
P47	CAAAATGGGATTGGGCGGCCCCCGCAAAAATAAC
P48	GTTATTTTTGCGGGGGCCGCCCAATCCCATTTTG
P49	CATAACTGAATTCATTAACACTAATGC
P50	CATGTAAATCACCCCTCAATAAATTATC
P51	GAGGGGTGATTTACATGGAAAATAATAAATGTAG
P52	GGGPTTATGCAATATAATCTATAG
P53	CAAATTTGGCATGTAAATCACCCCTC
P54	GAGGGGTGATTTACATGGAAAATAAAAAATGTTATTCAGAAGσσ
P55	GCAGGCAAGCTTTTATTTTCTACAGCAGTTAC

**qRT-PCR**	
RpoC qRT-PCR Fwd	CTAGCTGCTCCTATGTCTCACATC
RpoC qRT-PCR Rev	CCAGTCTCTCCTGGATCAACTA
CotA qRT-PCR Fwd	GCTGCATCTACTCCATTAGCAA
CotA qRT-PCR Rev	GCAATCATCACAATCGCAGT
CdeM qRT-PCR Fwd	TGTGGATGTTCAGATTCAGGAG
CdeM qRT-PCR Rev	TGCTAATGCCTCTTTCTTTTTC
CdeC qRT-PCR Fwd	AGCATCACCAAATCCAATCC
CdeC qRT-PCR Rev	AACTTGGCTTTCCACTTCCA

[a] underlined sequences represent introduced restriction sites.
