# Supplementary material for: A sporulation signature protease is required for assembly of the spore surface layers, germination and host colonization in Clostridioides difficile
Source: PLoS Pathog. 2023 Nov 13;19(11):e1011741. doi: 10.1371/journal.ppat.1011741 (PMC10681294; doi:10.1371/journal.ppat.1011741)
Supplement: Attachment — Submitted filename: Response to the reviewers.pdf [file ppat.1011741.s019.pdf]

August 30st, 2023

Dear Prof. McClane,

We have just completed the upload of the revised version of our manuscript, entitled "A sporulation signature protease is required for assembly of the spore surface layers, germination and host colonization in *Clostridioides difficile*" by Eleonora Marini, Carmen Olivença, Sara Ramalhete, Andrea Martinez Aguirre, Patrick Ingle, Manuel N. Melo, Wilson Antunes, Nigel P. Minton, Guillem Hernandez, Tiago N. Cordeiro, Joseph A. Sorg, Mónica Serrano and Adriano O. Henriques". The reference number for this manuscript is PPATHOGENS-S-23-00531-2.

Below, I list our answers to all the points raised by the reviewers. We found all the comments extremely useful, and in answering them, we believe that we have significantly improved the clarity and quality of our manuscript. Please note that several of the original authors are no longer in the group and that for the additional experiments we were asked to do I had to involve an additional researcher, who is now a co-author, Carmen Olivença.

We removed most of the conclusions and interpretations based on the AlphaFold model, in particular the issue of whether or not YabG has a catalytic triad. The similarity of the presumed active site to that of papain is no longer in the text, because, of the poor superimposition between the two, including the distance and orientation of the residues proposed in the original submission to form the catalytic center, as pointed out by one of the reviewers. We restrict our analysis mainly to the homologues of the residues that in the *Bacillus subtilis* protein were shown to form a catalytic dyad, although we comment on another residue required for activity. SuperPlots were adopted where appropriate and were a really nice addition. We have also condensed the manuscript, in particular the Discussion section. The Material and Methods that were previously included in the supplemental material, were transferred to the main text and condensed as much as possible. The main ideas included in the Supplementary Results and Discussion were also added, in a condensed form, to the main text. The Supplement now only includes Tables, Figures and Legends. Also please note that because part of the supplemental text on germination was transferred to the main text, a new section was created, entitled "The activity of YabG is required for proper spore germination" (lines 333-371).

Reviewer #1:

Weaknesses are minor. I think the importance and novelty of the study lies in what distinguishes YabG from C. difficile from that of B. subtilis, the authors might emphasize these differences, such as in the abstract.

Reply: we have done this in the abstract and also in the Discussion, as follows:

Abstract (lines 59-64 of the revised version, pdf file): "Thus, YabG also impinges upon the genetic program of the mother cell possibly by eliminating a transcriptional repressor. Although this activity has not been described for the *B. subtilis* protein and most of the YabG substrates

vary among sporeformers, the general role of the protease in the assembly of the spore surface is likely to be conserved across evolutionary distance."

Discussion (lines 781-787 of the revised version): "The control of the mother cell line of gene expression by YabG has not been reported for *B. subtilis*. In this organism, the mother cell line of gene expression is divided into several temporal and epistatic classes through the action of ancillary transcription factors that work together with σ^E (SpoIIID, GerR) and σ^K (GerE) [32,33,90–92]. Only SpoIIID is found in *C. difficile*. To what extent YabG influences gene expression in the mother cell and whether this role of YabG is unique to *C. difficile* remains to be studied".

Reviewer #2:

…The authors also identify a novel role for this morphogenetic protease in regulating the transcription of late sporulation genes, a finding that adds an important new layer to the regulation of C. difficile sporulation. While multiple nested transcriptional loops that control the timing and expression levels of late-acting sporulation factors to ensure the fidelity of the spore assembly process have been established in B. subtilis, there are only two known nested transcriptional loops in C. difficile: **SpoIIID in the mother cell and SpoVT in the forespore**. The authors' findings indicate that C. difficile has linked the activity of a morphogenetic protease to the assembly of robust spores that can withstand the harsh environment of the gut to establish colonization. This is a point that they could more explicitly state **in discussing the significance of their findings**. **By condensing their discussion of these transcriptional analyses, they would be able to more clearly and succinctly communicate the impact of this particular finding to the reader**.

We have now commented on this new layer of regulation that might be imposed by YabG, specifically on the mother cell line of gene expression, at the end of the Discussion (lines 781-787 of the revised version; see our answer to reviewer 1, above).

Indeed, while this work is thorough, carefully conducted, and clearly explained the manuscript would benefit from being condensed. There is too much speculation of the AlphaFold model, especially given that the model does not convincingly match their mutagenesis analyses (described in more detail below). Suggestions for condensing the manuscript are provided here:

- Combine Figures 1 and 2 and condense all the AlphaFold descriptions and interpretations
- Move 2A to the Supplement
- Move Figure 3 to the Supplement and condense the Discussion
- Move Figure 8 to the Supplement

Answer: This was done as suggested; Figures 1 and 2 were combined into what is now Figure 1. Panel A of figure 2 was moved to the supplement and it is now labeled as S5 Figure (please note that this figure has a new, additional panel, that was introduced in order to respond to a specific comment made by reviewer 1, relative to possible conserved cleavage sites between the YabG proteins from *B. subtilis* and *C. difficile* (see below).

Figure 3 is now figure S6. In the version previously submitted, figure 3 included data on the expression of *yabG*-SNAP at hour 20 of incubation in 70:30 medium whereas figure S5 included the same data but at hour 14 of sporulation. We now combined the two figures in one, which is now figure S6. Please also note that the data on the distribution of the signal intensity per cell is now represented as *SuperPlots* as suggested by reviewer 1 (see point below).

In what concerns Figure 8, however, we think its maintenance in the main text is justified the reason being that it makes an important point, *i.e.*, that the YabG-SNAP translational fusion is largely functional. This point was raised by reviewer 1 (see also below). This information was in the text before, but was now made more clear (lines 562-563): "As detailed below, the $YabG^{WT}$-$SNAP^{Cd}$ fusion is largely functional."

Also in lines 644-646: "Since full-length $YabG^{WT}$-$SNAP^{Cd}$ does not accumulate even in the ΔyabG mutant, it seems that the protein undergoes auto-proteolysis and thus, that the fusion protein is largely functional with respect to this activity (see also below).".

And in lines 657-662: "Importantly, the analysis of the cortex/coat/exosporium extracts by Coomassie staining and immunoblotting showed that the $YabG^{WT}$-$SNAP^{Cd}$ fusion, but not $YabG^{C207A}$-$SNAP^{Cd}$, restored both CotA and CdeM assembly and significant processing of Pre-pro-SleC to spores of a Δ*yabG* mutant and no other major differences to WT spores were noticed (Fig. 6B). We infer that with respect to the assembly of the spore surface, $YabG^{WT}$-$SNAP^{Cd}$ is largely functional.

The descriptions and interpretations related to the AlphaFold2 model were simplified and condensed.

In the results section, the detailed enumeration of the elements of secondary structure in SH3 and CheY-like domains, and in YabG were eliminated. The text now reads as follows:

In the results section, the detailed enumeration of the elements of secondary structure in SH3 and CheY-like domains, and in YabG were eliminated. The text now reads as follows: (lines 209-217 of the revised version): "The model generated for *C. difficile* YabG suggests that the protein consists of two independent structural domains, A (Fig. 1C, residues 1-57, in orange) and B (residues 99-286, in green) connected by a linker, L (residues 58-98, in blue) (see also S2. Fig). A search using the Fold Seek server [61] indicates structural similarity of domain A to proteins with an Src homology domain 3 (SH3), including for example the cyanobacterial protein PetP and *Escherichia coli* HspQ. PetP is a subunit of cytochrome *b6f* [62], while HspQ is both a substrate and a specificity and allosteric enhancing factor for the Lon protease [63,64] (S3 Fig). SH3 domains are small, 55-70 residues-long protein-protein interaction modules [65,66]. "

And for the CheY-like domain (lines 218-233): "Fold Seek also revealed structural similarity of B to the receiver domain of response regulators including, among the top hitters, the KdpE protein of *E. coli* (pdb identifier: 4I85), MicA from Streptococcus pneumoniae (7m0s), MtrA from *Mycoacterium tuberculosis* (3nhz) and CheY from Vibrio cholerae (4hnr) and *Thermotoga maritima* (4tmy). All of these proteins share the fold of the archetypal CheY response regulator

from E. coli [67–69]. The superimposition of the model obtained for domain B of YabG with the crystal structures of KdpE and CheY highlights the similarity to the CheY fold (S4 Fig). KdpE, MicA and MtrA have a receiver domain and a C-terminal DNA binding domain and function as transcription factors [70–72]. CheY, in turn, is a single domain response regulator which upon phosphorylation of an Asp residue, binds directly to the flagellar motor [67]. Most response regulators function through phosphorylation of a conserved aspartate residue [67–69] but others function independently of phosphorylation [68,69]. The phospho-acceptor residue in CheY is D57; this residue is not conserved in YabG with the homologous position occupied by Thr159 in *C. difficile* YabG and by Thr164 in the *B. subtilis* protein (Fig. 1B). Thus, YabG may function independently of phosphorylation."

Note that the description of the features known to be important for the regulation of the function of response regulators by phosphorylation, and that may be absent from the CheY-like domain of YabG were strongly dependent on the AlphaFold2 model and its interpretation. All this text was deleted, with the implication that the old Figure S1C was also eliminated.

In addition, the section called "**YabG uses a papain-like catalytic triad for auto-proteolysis**" was renamed to "**Residues of YabG involved in auto-proteolysis**". In fact, and as pointed out, the model does not show a good superimposition of the putative active site residues of YabG with those of papain and we now specifically say so. The text now reads as follows (lines 236-239 of the revised version): "The distances estimated between Cys207 and His161 from the model, and between the latter and Asp162 (Fig. 1D) are longer than in other Cys proteases and the side chain of His161 is not oriented towards Cys207 [58,59]."

- Discussion could be greatly condensed; speculation on YabG's catalytic center should be removed.

The Discussion was reduced/condensed; and the speculation on the catalytic center of YabG was removed. We just write about the inevitable comparison with the diad of *B. subtilis* YabG identified as C218/H172 (Yamazawa et al. 2022) (lines 698-704 of the revised section): "As found for the *B. subtilis* protein [52], C. difficile YabG also shows an auto proteolytic activity (Fig. 1E). Auto-proteolysis of B. subtilis YabG uses a C218/H172 catalytic dyad [52]. These residues are homologous to C207 and H161 of the *C. difficile* protein and may be directly involved in catalysis. Asp162, is also required for the activity of the *C. difficile* protein and is conserved among YabG ortologues but the role of the homologous residue in the *B. subtilis* protein, Asp173, has not been tested. In any event, C207, H161 and D162 are all located in the CheY-like domain B (Fig. 1C and D)".

The statements about the catalytic center were also tuned down in the Abstract. The text now reads as follows (lines 50-54): "the CheY-like domain carries residues required for activity, including Cys207 and His161, the homologues of which form a catalytic diad in the *B. subtilis* protein, and also Asp162. The substitution of any of these residues by Ala, eliminates an auto proteolytic activity as well as interdomain processing of CspBA, a reaction that releases the CspB protease, required for proper spore germination."

Also in the Author´s Summary, the text previously referring to a catalytic triad of a papain-like protease was eliminated. The text now reads as follows (lines 76-78): "The yabG gene, part of

a genomic signature for sporulation, codes for a cysteine protease, with residues required for catalysis embedded in a CheY-like response regulator receiver domain. “

And in the Introduction, the text now reads as follows. (lines 170-174): “Residues within the CheY-like domain are required for an auto-proteolytic activity that leads to its complete degradation and for cleavage of its substrates. These residues include C207 and H161 which occupy positions homologous to those shown to form a catalytic diad required for auto proteolysis in *B. subtilis* YabG [52].”

A key point that the authors should address in a revised manuscript is the clear substrate preference of YabG for cleavage after Arg residues. This has been shown with *B. subtilis* YabG (Yamazawa et al. 2022) as well as with *C. difficile* YabG’s substrates SleC and CspBA (Shrestha et al. 2019). **Are there arginine residues with YabG where it is likely autoprocessing as was observed for *B. subtilis* YabG?**

We have addressed this point (lines 262-269): “Auto-proteolysis of *B. subtilis* YabG leads to the accumulation of transiently stable fragments through cleavage after Arg5, Arg17, Arg49 and Arg93 [52]. Of these, Arg17 and Arg49 are conserved in C. difficile YabG (Arg9 and Arg41, respectively); the position homologous to Arg93 is occupied by a Lys in the *C. difficile* protein but there are two Arg´s in the vicinity of this residue (S5B Fig.). This suggests that the *C. difficile* protein may also be cleaved at least after Arg9 and Arg41. That *C. difficile* YabG is likely to be specific for Arg at the P1 position in line with cleavage of the SleC and CspBA after Arg residues [43]. “

Note that to support this new section of text, an amino acid alignment around the Arg17, Ar49 and Arg93 positions of of the *B. subtilis* protein with the homologous section of *C. difficile* YabG was produced and added to the new Figure S5 as panel B. The legend of the figure was also updated.

A detail that is missing from the Materials and Methods is **how many biological replicates were analyzed in their image quantifications**. **This information should be provided throughout the manuscript figure legends and in the Materials and methods**. Importantly, the use of Superplots would greatly increase the rigor of their data analysis. If only one biological replicate was quantified, this should be indicated. Notably, no statistical analyses can be performed on one replicate even if hundreds or thousands of measurements were made for an n=1 sample. If multiple biological replicates were quantified, the statistical analyses should be performed on the three replicate values rather than the number of events quantified. https://rupress.org/jcb/article/219/6/e202001064/151717/SuperPlots-Communicating-reproducibility-and

The use of SuperPlots was adopted. They were used for **1**) the quantification of the single cell data on the expression of a transcriptional yabG-SNAP fusion, both to correlate expression with stages of morphogenesis and between the WT and a *sigK* mutant (S6 Fig), **2**) to quantifiy the fluorescence intensity of the polar region of spores stained with FM4-64 (S11 Fig), **3**) to compare the expression between the WT and a *yabG* mutant (S14 Fig) was adopted. Both the Material and Methods and the figure legends were updated accordingly. The article indicated by the reviewer (Lord et al., 2020) was also added to the reference list.

Material and Methods (lines 976-979): “The intensity of the FM4-64 signal in the spore polar appendage was quantified using ImageJ (http://rsbweb.nih.gov/ij/) by drawing a 0.25 µm diameter circle in the appendage region. The data from three independent experiments was represented using SuperPlots [103]”.

Legend for figure S6 (lines 1536-1542): “SuperPlots were used to represent the data from three biological replicates; each dot corresponds to one cell, color-coded by experiment. The large circles represent the means from each experiment which were used to calculate the mean and standard error of the mean (horizontal lines) for the ensemble of the three experiments. Statistical analysis was carried using a Student’s t test (right panels) or an one-way ANOVA followed by Tukey’s multiple comparations test (left panels). *, p<0.05; ***, p< 0.0001.”

Legend for Figure S11 (lines 1614-1618): “SuperPlots were used to represent the data; each dot corresponds to one cell, color coded by experiment. The large circles represent the means from each experiment which were used to calculate the mean and standard error of the mean (horizontal lines) for the ensemble of the three experiments. Statistical analysis was carried out using using ANOVA and Tukey’s test. *, p<0.05; **, p< 0.001.”

Legend for Figure S14 (lines 1655-1660): “SuperPlots were used to represent the data, with each dot corresponding to one cell and the three experiments shown with different colors. The large circles represent the means from each experiment and were used to calculate the mean and standard error of the mean (horizontal lines) for the collective of the three experiments. Statistical analysis was carried out using a Student’s t-test. * indicates p<0.05.”

Part II – Major Issues: Key Experiments Required for Acceptance

Reviewer #1: I do not believe additional experiments are needed.

Reviewer #2: My most significant concern with the manuscript is that the AlphaFold model shown in Figures 1D and S4 **do not show an active site with the catalytic residues aligned in a manner that is consistent with most proteases**. The catalytic residues of papain are within much closer proximity than the distance between the likely catalytic residues determined experimentally and via sequence alignment. **The putative catalytic Aspartate 162 is even oriented away from the catalytic His161 and Cys207 residues**. Thus, language like “Cys 207, His161, and Asp162 can be superimposed onto the catalytic triad” is not reflected in the models shown. Given that the AlphaFold model does not give an accurate prediction of the active site, the proposed similarity of YabG to the receiver domain of response regulators is less convincing, **especially since it does not appear to be regulated by phosphorylation and the authors do not perform structure-guided mutagenesis of the different domains**.

As detailed above, we have eliminated the speculations on the hypothetical catalytic. Triad of YabG, its superimposition with the catalytic center of papain and we now specifically comment of the distance and orientation of the proposed catalytic residues. So these points were addressed. We think, however that the overall fold of the (catalytic) domain B is that of a receiver domain such as that of PetP and CheY as shown in figure S4. The model may not be accurate in details related to the orientation of the side chains of residues close to the surface of the molecule as those proposed to be catalytic, but the overall statistics, included in figure

S2 are good and together with the FoldSeek clearly suggest a CheY-like fold. However, the considerations relative to the orientation of specific side chains may have also biased our analysis of the presence/absence of additional features of receiver domains. Specifically, although the lack of conservation of the phosphoryl acceptor residue (absent from both the YabG proteins of *B. subtilis* and *C. difficile*) is still presented, the extensive text on all the other features was removed. In addition, panel B of Figure S1, which serve as a support for that part of the text was eliminated. Structure-based mutagenesis to test some of those ideas is under way.

For the fractionation experiments, the authorshe features indicate that their two fractions represent the coat/exosporium and cortex/cytosol. However, it is likely that their coat/exosporium fraction likely contains cortex proteins. The authors should blot for SleC to define their fractions, since C. difficile SleC is the sole protein known to be localized specifically to the cortex layer (Baloh et al. 2022).

The comment refers to figures 4 (which is now Fig. 2) and 8 (which is now Fig. 6). We have repeated the experiments three times and included a panel in which the different fractions obtained from purified spores were subject to immunoblotting with anti-SleC antibodies. SleC (both the full-length form and the processed form) was detected in what we called the coat/exosporium and the core/cortex fractions. Therefore, and as suggested by the reviewer, our “coat/exoporium” fraction may contain cortex-associated proteins, at least SleC.

Because of this, the coat/exosporium fraction was renamed cortex/coat/exosporium fraction in the figures and throughout the text, methods included. Also, Baloh et al (2022) the publication where SleC is localized to the cortex, was added to the reference list.

Specifically, in the Results section, the following changes were made (lines 374-378): “Spores of the four strains were purified on gradients of metrizoic acid, and the proteins extracted in a buffer with SDS and reducing agents; the resulting spores were then treated with lysozyme and proteins again extracted (see the Material and Methods). This produced a cortex/coat/exosporium fraction and a core/cortex fraction (as explained below).”

Also in the Results section (lines 410-413): “As expected, the spore core protein GPR was detected by immunoblotting only in the core/cortex fraction (Fig. 2A). SleC, however, shown before to be associated with the cortex [74] was detected in the cortex/coat/exosporium fraction of WT or *yabG*^{C} spores, mostly in its processed form (Fig. 2A).”

Further down (lines 418-421): “While enriching for coat/exosporium proteins, which are not detected in the core/cortex fraction, our extraction procedure also releases cortex-associated proteins, at least SleC. That proteins known to be associated with the cortex are extracted with the coat/exosporium has been reported before [81]”

And further down (lines 655-657): “As above (Fig. 2A), SleC was only detected in the cortex/coat/exosporium fraction [74], whereas GPR was only detected in the core/cortex fraction (Fig. 6B).

The Methods also refer to the cortex/coat/exopsoirum and core/cortex fractions (lines 926-931): “The suspension was boiled for 5 minutes and the spores collected by centrifugation. The supernatant, corresponds to a cortex/coat/exosporium fraction. The spore sediment was washed twice with PBS with 0.1% Tween-20, and incubated with 50mM Tris-HCl pH 8 with 2mg/ml lysozyme for 2 h at 37 ºC to digest the spore cortex peptidoglycan and release core/cortex-associated proteins.”

The authors should formally report whether their **fluorescent-protein tagged YabG constructs are functional**.

This is a really important point and the reason why we maintained the former Figure 8 (now Figure 6) in the manuscript. YabG was tagged with the SNAP-reporter and our conclusion is that the fusion protein is largely functional. We refer to this in the Results section, in two occasions.

First, in the section entitled “**YabG localizes asymmetrically in mature spores”** (lines 644-646): “Since full-length YabG^{WT}-SNAP^{Cd} does not accumulate even in the Δ*yabG* mutant, it seems that the protein undergoes auto-proteolysis and thus, that the fusion protein is largely functional with respect to this activity (see also below).”

Also in lines 657-662 (end of the section): “Importantly, the analysis of the cortex/coat/exosporium extracts by Coomassie staining and immunoblotting showed that the YabG^{WT}-SNAP^{Cd} fusion, but not YabG^{C207A}-SNAP^{Cd}, restored both CotA and CdeM assembly and significant processing of Pre-pro-SleC to spores of a Δ*yabG* mutant and no other major differences to WT spores were noticed (S6B Fig.). We infer that with respect to the assembly of the spore surface, YabG^{WT}-SNAP^{Cd} is largely functional.”

Part III – Minor Issues: Editorial and Data Presentation Modifications
Please use this section for editorial suggestions as well as relatively minor modifications of existing data that would enhance clarity.

Reviewer #1: Can YabG be attributed a catalytic triad **if one of the triad residues is dispensable for activity**?

As assessed using the auto-proteolysis assay, Cys207, His162 and Asp161 are all required for activity. This prompted us to speculate, in the version originally submitted that YabG could have a catalytic triad. This was based on the idea that three amino acids, Asp161, H162 and Cys207 superimposed well on the catalytic triad of papain. This was wrong and we removed all this material from the text (see our response to comments above). We now write about a catalytic dyad because C218 and H172, homologous to Cys162 and His161 of the *C. difficile* protein, are required for auto-proteolysis. We no longer write about a triad. However, we made two other Ala substitutions, of Asp161 (adjacent to the putative catalytic His162) and of Asp248 (not in the same region of the protein, and served sort of a control). The Asp161Ala substitution prevented activity; the Asp248Ala did not (Figure 1E). In brief, we no longer discuss a triad, but yes, there is one Asp residue, Asp161 that is required for activity by an unknown mechanism while another, Asp248 is **dispensable** for activity.

Lines 447-450, are these percentages relative to total or to the number of spores with polar appendages? Also, I suggest putting the information on adherence of the coat to the cortex together, i.e. the information in lines 450-452 and 462-463 should be in the same paragraph for easier comparison

The percentages are relative to the number of spores with polar appendages. This was clarified in the text (lines 472-473): "Importantly, in about 35% of the *yabG*^{C207A} spores with appendages, …"

Also, the text was changed as suggested and the information referred to by the reviewer is now in the same paragraph (lines 484-486): "…which may be shared by the rest of the exosporium. Consistent with the absence of CdeM from the extracts of *yabG* spores (above, [24]), the exosporium-like layer is often absent …"

In the animal experiments, there appear to be few animals in the group infected with the mutant. This does not appear to be due to animal survival since there are no differences in survival between the WT and mutant infected groups. Is it that there are mutant-infected animals with CFU below the limit of detection? If so, were those data included in determination of medians?
Animal experiment data – please indicate what is shown in Fig 9B. Presumably the bars represent medians. **What is the limit of detection**?

The data has been set to the median values and the limit of detection is 1,000 CFU / g. This has been updated on the graph. The limit of detection is also indicated in the new version of the graph. Some animals in the analysis that did not give a fecal sample because they were sick, so they are not included. The text now reads as follows (lines 679-683): "We note that the number of animals infected with ΔyabG spores is lower that for the WT because some of those animals were sick and did not produce give a fecal sample; for this reason they were not included in the analysis. In any case, *yabG* seems to have an important role during colonization of the hamster colon. "

Also in the legend for figure 7B (Figure in the original submission) the detection limit was included (lines 1463-1465): "Faecal samples were collected daily, and both *C. difficile* vegetative cells and spores were enumerated by plating. The data was set to the median values and the limit of detection (dashed line) is shown.".

How many independent samples/cells were analyzed by immunoblot and microscopy? This information is needed so the reader can evaluate the representativeness and accuracy of percentages stated in the text.

This was carefully revised throughout the text. The number of cells and the number of independent experiments is now referred to in all relevant figure legends and in some cases also in the Material and Methods section. All microscopy experiments and immunoblots (and in general all the experiments reported in the manuscript) were done independently at least three times, with the exception of the transmission electron microscopy which was done only twice. Also note that SuperPlots, which imply three biological experiments, were adopted in as suggested below.

Consider the content of the materials and methods in the main paper versus supplemental. For example, I think experimental parameters for microscopy are more important in the main text than strain generation.

All of the Material and Methods are now part of the main text, as also suggested by the Editor, in conformity to the rules of the journal. The text was also shorted as much as possible.

Other minor points/recommended edits:
Please remove superfluous commas throughout, and there are multiple places where commas are needed for clarity.

The text was carefully revised with respect to the use of commas.

L95, recommend changing to "certain bile salts"

The text was changed as suggested. It now reads as follows (lines 96-98): "Infection starts with the ingestion of spores that will germinate in the small intestine in response to certain bile salts; at least a fraction of the vegetative cells that outgrow from spores…"

L97, differentiate into spores

This was corrected (lines 97-99): "…a fraction of the vegetative cells that outgrow from spores will produce the TcdA and TcdB toxins, and some will differentiate into spores..."

There are numerous typos and other errors in the Discussion that need correcting, including but not limited to lines 708, 710, 727, 729, 775, 811

All these typos were corrected and the text carefully revised (also with respect to the use of commas as suggested above).

L164, "mutations in yabG were found to render…"

The text was changed and now reads as follows (lines 167-168): "…and mutations in *yabG* were found to render germination in response to the bile salt taurocholate independent of co-germinants such as glycine [43].".

L166, typo

This was corrected; "germinqnst" was changed to "germinants" (line 168 of the revised version).

L193, "activity that causes"

This was corrected (lines 192-194): "… *B. subtilis* YabG is a cysteine protease and suggested that it uses a catalytic dyad formed by Cys218 and His172 in an auto proteolytic activity that causes its rapid degradation…".

L212-216 – sentence is difficult to follow. Recommend removing all words after HspQ or breaking up the sentence into two

The sentence was changed for clarity. The text now reads as follows (lines 212-217): "A search using the Fold Seek server [61] indicates structural similarity of domain A to proteins

with an Src homology domain 3 (SH3), including for example the cyanobacterial protein PetP and *Escherichia coli* HspQ. PetP is a subunit of cytochrome *b6f* [62], while HspQ is both a substrate and a specificity and allosteric enhancing factor for the Lon protease [63,64] (S3 Fig). SH3 domains are small, 55-70 residues-long protein-protein interaction modules [65,66].”

L240-250 – the organization of this section makes it difficult to follow. Suggest reorganizing to more explicitly delineate what is and is not similar to CheY

In response to other comments related to speculation based on the AlphaFol2 model of YabG, this part of the text was deleted in the revised version.

L266, recommend removing “using auto-induction” since expression appears to be under the control of a T7 promoter

Production of is in fact under the control of a T7lac prmoter, but expression is achieved in a medium that allows for induction in the absence of IPTG addition. We clarified this sentence and added a reference (lines 243-245): “To test whether *C. difficile* YabG showed auto-proteolytic activity and if so, what residues of the putative active site were involved, we overproduced YabG^{WT} in *E. coli*, using an auto-induction regime [73], as a N-terminal His$_{10}$ fusion.”

Also, in the first section of the material and methods, the text now reads as follows (lines 829-831): “The *Escherichia coli* strain DH5α (Invitrogen) was used for molecular cloning and BL21(DE3) (Novagen) was used for the over-production of WT His$_{10}$-YabG and its variants and CspBA-Strep-tag by auto-induction [73];”.

Fig 1 – consider swapping the order of the 2nd and 3rd alignments in panel B so that they are in numerical order

This was done as suggested.

Fig 4C, the arrows in the bottom panels look orange/brown, but text states they’re black

Figure 4 is now figure 2; the arrowheads that the reviewer refers to were changed to black; also in the related figure S12.

Fig 5C – statistical analysis is needed for the qRT-PCR data

The statistical analysis was added to the figure and specified in the figure legend (lines 1407-1412): “Quantification of the expression of the indicated genes (*cdeC*, *cdeM* and *cotA*) by qRT-PCR. Total RNA was extracted from *C. difficile* 630Δerm and Δ*yabG* strains grown in 70:30 agar plates for 14 and 20 hours. The graph shows the fold change in the expression of *cdeC*, *cdeM* and *cotA* between the Δ*yabG* and the WT. Error bars correspond to the standard deviation derived from three biological replicates. Statistical analysis used a Student’s t-test: * $p<0.01$; ** $p<0.001$.”

Text in lines 803-806 should be placed earlier in this section

Text was changed and its placement too, as suggested (lines 613-616). “In any event, since YabG^{C207A}-SNAP^{Cd} localizes earlier than the WT, the catalytic activity of YabG controls, at

least in part, the localization of the protein during sporulation. In that sense, assembly of YabG is auto-regulatory." However, other changes were made to this section of the text in response to other comments.

L813, reword to "This reduced germination might…"

This part of the text rearranged in response to other comments and the suggestion no longer applies.

L821, "…CotE contributes to…"

This section of the original text is not in the revised version.

Reviewer #2:

Line 87: please add a period after "therapy"

This was corrected (lines 88-89): "…*Clostridioides difficile* [1] is the leading cause of nosocomial diarrhoea linked to antibiotic therapy. Infection can, however…"

Line 94: please revise to indicate that outgrowing cells are generated from germinating spores

The text was changed and now reads as follows (lines 97-98): "…at least a fraction of the vegetative cells that outgrow from spores will produce the TcdA and TcdB toxins…":

Line 122: Consider changing "and" to ", which"

The suggestion was followed. The text now reads as follows (lines 97-98): "…at least a fraction of the vegetative cells that outgrow from spores will produce the TcdA and TcdB toxins".

Line 123: please delete the comma after "goal"

This was corrected (lines 123-126): "Clearly, the identification and functional characterization of proteins that govern assembly of the coat and exosporium layers remains an important research goal that will inform us on the role and behaviour of spores during the initial stages of infection..".

Line 143: please add a comma after "shown"

The sentence was altered for clarity and the change is no longer justified. The text now reads as follows (lines 145-146): "…and in *C. difficile*, *yabG* plays a part in spore germination ([42,43]; see also below).".

Line 146: change "assembly" to "assembling"

This was done as suggested (lines 144-146): "In *B. subtilis* and in *B. anthracis*, a role for *yabG* in the assembly of the spore coat has been shown [36–41] and in *C. difficile*, *yabG* plays a part in spore germination ([42,43]; see also below)…".

Line 155: please add "accumulate in their unprocessed forms"

This was done (lines 157-159): "Two other proteins, not found in *Bacillus* sporeformers, also accumulate in their unprocessed forms in spores of a *C. difficile yabG* mutant, CspBA and Pre-pro-SleC [42,43, 44]."

Line 166. It would be helpful to add a sentence describing the proposed role of CspA in sensing co-germinant.

This was done (lines 166-168): "CspA is important for recognition of co-germinants and mutations in *yabG* were found to render germination in response to the bile salt taurocholate independent of co-germinants such as glycine [43]."

Line 172: consider condensing "analysis of an in-frame…" to "Loss of YabG or its catalytic activity alters the assembly of the exosporium

This was done (lines 367-369): " Thus, the partial germination defect of Δ*yabG* and yabG^{C207A} spores may be due, at least in part, to loss of YabG activity, which in turn impairs the release of CspB from CspBA and the production of pro-SleC."

Line 174: YabG is important for regulating the sensitivity of C. difficile spores to "co-germinants"

The sentence was changed and now reads as follows (lines 175-179): "We show that YabG governs attachment of the coat to the underlying cortex peptidoglycan, formation of the exosporium and is also involved in germination in line with its recently demonstrated role in regulating the sensitivity of *C. difficile* spores to co-germinants [43]."

Lines 175-177: The sentences starting with "These phenotypes" could be removed.

The sentence was removed as suggested.

Line 197: remove the comma before "reveals"

This was corrected (lines 197-198): "An alignment of the residues around Cys218 of the *B. subtilis* YabG protein with the corresponding region from selected orthologues, reveals a highly conserved region…"

Line 223: "pneumoniae" is misspelled
This was corrected (lines 219-220): "…the KdpE protein of *E. coli* (pdb identifier: 4I85), MicA from *Streptococcus pneumoniae* (7m0s), MtrA from

Line 286: it is unclear whether separation of CspB and CspA is required for germination because even in the absence of YabG or if YabG-dependent cleavage sites, CspBA still undergoes processing at aberrant sites.

The processing site or sites of CspBA are not know for sure and to comment on this would take us well away from the main point. So we thought it would be better not to speculate about this point. The text, however, was changed for clarity (lines 273-275): "YabG was found to be involved in CspBA processing [42,43]. To assess the role of Cys207 in the reaction, YabG^{WT} or YabG^{C207A} were co-produced in *E. coli* together with CspBA."

Line 729: add a period after “YabG” and “The”

This part of the text was deleted in the revised version and the correction no longer applies.

Line 1294: change “unspecific” to “non-specific”

This was corrected, also elsewhere in the text. The text now reads as follows (line 1369-1372): “The green arrowhead in **F** shows the position of CspB released from CspBA, independently of YabG, while the red arrowhead points to CspB released from CspBA through the action of YabG. The asterisk refers to an non-specific band.”

Line 1314: “not” should be changed to “no”

This was corrected (line 1535): “…No signal was detected in *sigE*::*ermB* sporangia”

Line 1325: please add a comma before “while”

This was corrected (lines 1379-1381): “The red arrowheads indicate proteins that appear to be more extractable from Δ*yabG* and *yabG*^{C207A} spores while black arrowheads show proteins with reduced extractability.”

Line 1354: Please change “Total RNAs was” to “Total RNA was extracted”

This was corrected (lines 1408-1409): “ Total RNA was extracted from *C. difficile* 630Δ*erm* and Δ*yabG* strains grown in 70:30 agar plates for 14 and 20 hours.”

Please note that “phase-dark”, “phase-bright”, “phase-contrast”, “fold-change,” should be hyphenated.

This was corrected throughout the main text and figure legends.

I hope I have addressed all the questions raised in a satisfactory way, but please let me know in case you need any point to be further clarified.

Sincerely yours,

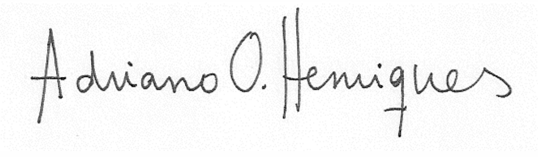

Adriano O. Henriques, PhD
Associate Professor
Instituto de Tecnologia Química e Biológica
Universidade Nova de Lisboa
Phone: 351-21-4469521; *Email*: aoh@itqb.unl.pt
